# Beyond the herd: investigating livestock feeding strategies in the Iron Age Iberian Peninsula (3rd c. BC) through a multi-isotope analysis

**DOI:** 10.1007/s12520-024-02116-z

**Published:** 2024-12-26

**Authors:** Chiara Messana, Carlos Tornero, Lídia Colominas

**Affiliations:** 1 Institut Català de Paleoecologia Humana i Evolució Social (IPHES-CERCA), Tarragona, ES Spain; 2 Departament d’Història i Història de l’Art, Universitat Rovira i Virgili (URV), Tarragona, 43002 ES Spain; 3 Department of Prehistory, Autonomous University of Barcelona (UAB), Bellaterra, ES Spain; 4 Institut Català d’Arqueologia Clàssica (ICAC-CERCA), Tarragona, ES Spain

**Keywords:** Animal diet, Stable isotopes, δ^13^C and δ^18^O tooth bioapatite, Bone collagen δ^13^C and δ^15^N values, Iron Age communities, NE Iberian Peninsula

## Abstract

During the Iron Age, north-eastern Iberian communities relied on crop cultivation and animal husbandry for their subsistence. The latter was mainly focused on caprine, with sheep being prominent due to their suitability to the Mediterranean climate, orography, and environment. Despite the pivotal role of sheep in livestock husbandry, information on Iberian communities’ feeding strategies for this species is limited. To address this lacuna, this study investigates the livestock feeding habits at four Catalan sites dating back to the 3rd century BC: Mas Castellar de Pontós, Tossal de Baltarga, Sant Esteve d’Olius, and Turó de la Rovira. Bulk carbon and nitrogen isotope analyses (δ^13^C and δ^15^N) of bone collagen and sequential carbon and oxygen analyses (δ^13^C and δ^18^O) of dental enamel were performed on 74 remains (43 bones, 31 teeth) from 51 sheep. Additionally, 153 bone remains from other domestic and wild species from the four sites were analysed. Our results revealed the complex and adaptive livestock feeding strategies of Iron Age Iberian populations. Sheep exhibited minimal diet variations, mainly consuming C_3_ plants across the four sites. Nevertheless, their feeding habits changed seasonally depending on pasture availability and settlement requirements. The other main domestic species were subject to individualised and variable feeding strategies within the same settlement. This diversified feeding management unveiled the complex and adaptive nature of the husbandry practices employed by Iron Age Iberian communities. Finally, the extensive number of remains analysed provides the first data on livestock feeding strategies for the Middle/Late Iron Age in the north-eastern Iberian Peninsula.

**Supplementary Information:**

The online version contains supplementary material available at 10.1007/s12520-024-02116-z.

## Introduction

### Setting the stage: historical context and aim of the research

The Iron Age Iberian communities in the north-eastern Peninsula were organised into hierarchical proto-state structures (Gracia 2005; Almagro-Gorbea 2014; Sanmartí 2014, 2021). The settlement pattern was organised through a network of interconnected open-air centres, developed around central nuclei, the so-called first-order cities. These settlements included fortified centres, rural establishments, small villages, and silo fields, each with specific functions (Gracia 2005; Sanmartí 2014, 2021). The subsistence strategies of these communities were based on peasant economies. Cereal agriculture was the main source of subsistence. In the north-eastern Iberian Peninsula, crop cultivations were dominated by wheat (*Triticum aestivum/durum*) and barley (*Hordeum vulgare*), supplemented by millet (*Panicum miliaceum*) and leguminous plants *(Lens culinaris*, *Pisum sativum*, *Vicia faba*) as secondary crops (Alonso 2000; Riera et al. 2020). A high level of complexity in crop management has been attested, including potentially the use of rotation cycles in cultivation practices (Alonso 2000; Riera et al. 2020). In addition to crop cultivation, herding practices were also a fundamental activity to ensure both the growth and sustainability of these Iron Age communities.

Over two decades of zooarchaeological research have allowed the characterisation of livestock husbandry during that time and in that region. Livestock management included diversification in the exploitation of different domestic species (sheep, goats, cattle, pigs, dogs, horses, rabbits, and chicken), allowing the reduction of dependence on a single species and the obtaining of diversified animal resources (Franquesa et al. 2000; Colominas 2006). In this scenario of self-dependence, sheep was the most important species for the economies of the time, as a result of its adaptation to the Mediterranean climate and environment (Miró and Molist 1982a, b; Barbera et al. 1986; Casellas 1993; Albizuri and Nadal 1999; Franquesa et al. 2000; Perez et al. 2007; Colominas 2008, 2013a). This phenomenon can be traced back to the first introduction of this taxon in the Iberian Peninsula during the Early Neolithic, around the VI millennium cal BC, and persisted in subsequent periods before the Iron Age (Saña et al. 2000; Nieto et al. 2021).

Recently, new biomarker studies have provided information on sheep management during this period that is difficult to obtain through traditional zooarchaeological studies. Isotopic oxygen and carbon analyses on sheep molars have documented the complex and diversified husbandry strategies adopted by the Iron Age communities in the north-eastern Iberian Peninsula. When necessary, shepherds intentionally manipulated sheep births, controlling both the seasonality and the duration of the lambing period (Messana et al. 2023a). Further, the use of multiple isotopic markers has allowed the investigation of herd management mobility patterns (Valenzuela et al. 2016, 2018;), even revealing the existence of altitudinal movements already during this period (Messana et al. 2023b). In both cases (management of sheep reproduction and mobility), the studies show the dependence on the environmental, climatic, economic, and regional context in which livestock farming was practised, documenting differences in the techniques and strategies developed depending on the location of the settlement analysed.

**Fig. 1:**
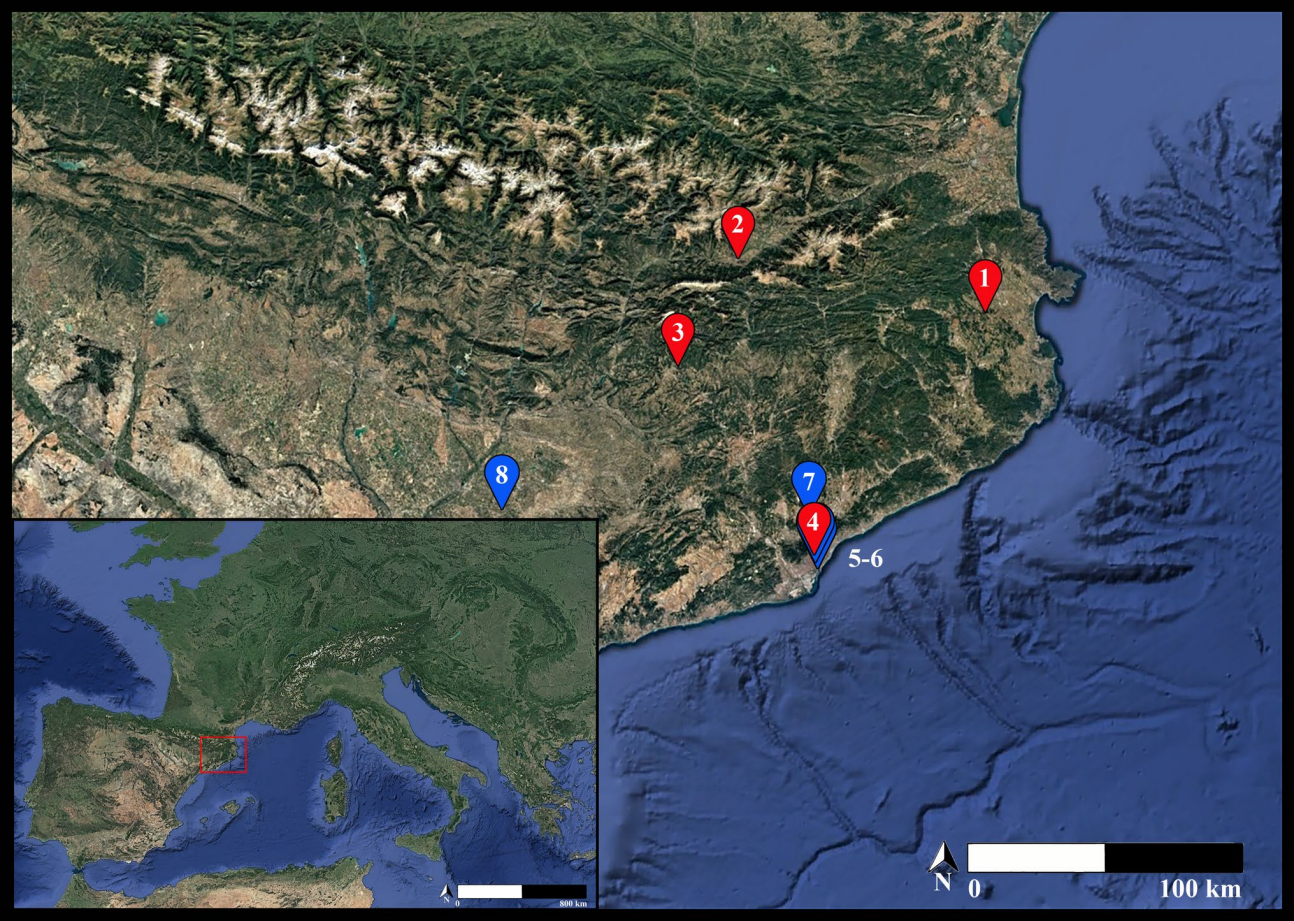
Geographical positions of the four Iberian sites (in red; (1) Mas Castellar de Pontós, (2) Tossal de Baltarga, (3) Sant Esteve d’Olius, and (4) Turó de la Rovira) and the comparison sites from the literature (in blue; (5) Vila de Madrid, (6) Carrer Ample 1, (7) Can Roqueta, and (8) Minferri) included in this research (Google Earth Pro modified)

This paper investigates the management of sheep feeding, an aspect for which there is no previous information, and which is essential for the exploitation of domestic animals. To achieve this, a biogeochemical approach (stable isotopes) is applied to investigate the feeding strategies employed by shepherds in the north-eastern Iberian Peninsula. Carbon and nitrogen isotope analysis (δ^13^C and δ^15^N) of collagen was performed on the bone remains of the main domestic and wild species recovered from four Middle/Late Iron Age settlements: Mas Castellar de Pontós (MC), Tossal de Baltarga (BTB), Sant Esteve d’Olius (O), and Turó de la Rovira (TR). Our results are compared with existing data from sites located in the study area and dating from earlier and later periods: Bronze Age (BA), Iron Age (IA), and Roman Period (RP; Fig. 1). In addition, a portion of the analysed sheep remains was also sampled for the sequential analysis of carbon and oxygen values (δ^13^C and δ^18^O) of dental enamel.

The available data on the dietary signals of livestock during the Protohistoric periods in the Iberian Peninsula are limited. Indeed, this research represents the first large-scale study of livestock feeding strategies during the Middle/Late Iron Age in the north-eastern Iberian Peninsula. Furthermore, the significant number of individuals and species here analysed could serve as a reference for future research in the same study area.

### Principles: stable isotopes and herding diet

Stable isotope analysis has proven to be a reliable and valuable tool for reconstructing an individual’s diet, by measuring the biochemical composition of preserved body tissues in the archaeological record. Isotopic composition of food and water ingested by an animal is recorded in its bones and teeth. The δ^13^C and δ^15^N isotopic values from bone collagen provide a quantitative assessment of the protein components of an individual’s diet, corresponding to the average food intake during the last 10–15 years of life. This duration corresponds to the time it takes, approximately, for bone collagen to remodel itself during an individual’s lifetime (Ambrose 1986; Tykot 2004; Hedges et al. 2007). The isotopic signatures recorded in animal tissue differ from those of the initial source consumed as a result of isotopic fractionation. The carbon isotopic fractionation between dietary intake and the consumer is + 5‰, and + 1‰ from collagen diet to collagen consumer along the trophic chain (DeNiro and Epstein 1978; Schoeninger and DeNiro 1984; Ambrose and Norr 1993; Bocherens and Drucker 2003). The nitrogen isotopic fractionation, on the other hand, is between 3–5‰ at each trophic step (Minagawa and Wada 1984; O’Connell et al. 2012; Matsubayashi and Tayasu 2019). The δ^13^C value measured in bulk collagen derives from the isotopic composition of the plants consumed by an individual. This varies according to the photosynthetic pathways of the plants, i.e. C_3_ e C_4_ (Bender 1971; Farquhar et al. 1989). The average δ^13^C value expected for C_3_ plants is −28.5‰, ranging from − 20‰ to −37‰, with − 23‰ as the maximum value recommended for typical C_3_ plants. On the other hand, the average δ^13^C value for C_4_ plants is −13‰, ranging from − 10‰ −14‰. Extremely low δ^13^C values indicate exclusive consumption of plants grown in closed-canopy forests, with a cut-off of −31.5‰ (Tieszen and Button 1989; Tieszen 1991; Kohn and Cerling 2002; Kohn 2010). These values have been calculated in modern plants, whereas for those from pre-industrial times, a correction of + 1.5‰ is required to compensate for the fossil fuel effect (Friedli et al. 1986; Marino and McElroy 1991; Feranec and MacFadden 2006). In this study, the threshold above which δ^13^C values are considered indicative of a complement of C_4_ plants in an individual’s diet is set around − 23‰ (~ −17‰ in collagen consumers and ~ −7.4‰ in the dental enamel of large ruminants; Fabre et al. 2023; Ramírez-Pedraza et al. 2023). C_3_ plants dominate the Western European vegetation, whilst wild C_4_ plants are either inexistent or residual in temperate environments (Mateu 1992; Pyankov et al. 2010). Nevertheless, a small portion of wild C_4_ plants, particularly halophytic communities, is attested in the marshy areas of the Iberian Mediterranean coast (Molina et al. 2003; Gestí 2006; Casals 2007; Pla de Ports de Catalunya 2007–2015; Ejarque et al. 2016; Valenzuela-Lamas et al. 2016; Salazar-Mendías and Lendínez 2020). Therefore, the presence of C_4_ plant intake in the studied chronotopic context suggests that animals likely consumed cultivated C_4_ plants, such as millet and foxtail millet. However, the possibility that livestock may have consumed wild C_4_ plants from coastal areas cannot be entirely ruled out. The carbon isotopic signature in collagen also allows discrimination between a diet primarily comprised of either terrestrial or marine foods. Indeed, marine plants exhibit higher values not only of carbon but also of nitrogen (Richards and Hedges 1999; Tykot 2004; Fisher et al. 2007).

The isotopic nitrogen composition in terrestrial plants mainly depends on the type of nitrogen obtained and the way it is absorbed from the substrate. This can occur either through fixation mediated by symbiotic microbes or directly from soil nitrates (Evans 2001; Tykot 2004; Szpak 2014; Katzenberg 2018). The δ^15^N values measured in collagen are used to ascertain the trophic level of an individual within the food web. Indeed, there is a ^15^N enrichment through successively higher trophic levels. Consequently, the δ^15^N isotope values of predators are higher than those of their prey (DeNiro and Epstein 1978; Schoeninger and DeNiro 1984; Katzenberg 2018). Nevertheless, soil ^15^N enrichment can vary as a result of both natural factors (substrate type, degree of soil development, nutrient availability, mycorrhizal associations, soil associations, climate) and anthropogenic ones (i.e. manuring; Bogaard et al. 2007; Fraser et al. 2011; Szpak 2014; Jordana et al. 2019; Makhad et al. 2022). Consequently, when comparing individuals from different ecosystems, it is important to consider the isotopic composition of the local food web (O’Connell et al. 2012; Katzenberg 2018). For this reason, it is essential to create a local baseline that enables a correct interpretation of the nitrogen isotope values of each individual.

Once mineralised, dental enamel does not remodel. As a consequence, the mineral fraction of the enamel (i.e. bioapatite compound) reflects the isotopic signature recorded during its formation. In sheep, the crown formation of the second molar (M_2_) begins between the first and second month and is completed at 12 months. On the other hand, the third molar (M_3_) starts forming from the tenth month onwards and is completed at around 22 months (Milhaud and Nézit 1991; Hillson 2005; Zazzo et al. 2010). However, there is a delay in the duration of the enamel mineralisation process, which is estimated to be around 6 months (Zazzo et al. 2010; Blaise and Balasse 2011; Balasse et al. 2012). Each of the two molars individually allows the reconstruction of one year of an individual’s life. The information obtained from the combination of the two molars makes it possible to trace back the first two years of a sheep’s life. Therefore, the sequential δ^13^C and δ^18^O analysis of dental enamel provides a snapshot of an individual’s diet and pasture environment during the period of tooth formation. The δ^13^C values measured in dental bioapatite are related to the carbon isotopic composition of the plants ingested by an individual (Lee-Thorp and Van de Merwe 1987). Large ruminant mammals exhibit a ^13^C enrichment of 14.1 ± 0.5‰ between diet and enamel bioapatite (Cerling and Harris 1999). The oxygen isotope values recorded in bioapatite are mainly derived from ingested water and consumed plants (Longinelli 1984; Luz et al. 1984). These isotopic signatures are related to meteoric water and temperature, which vary seasonally at temperate latitudes. As a result, at mid and high latitudes, higher δ^18^O values reflect the warm season, whilst lower δ^18^O values are recorded during the cold season (Gat 1980). It is, therefore, possible to detect changes in an individual’s diet during the first years of life with a seasonal resolution by combining the δ^13^C and δ^18^O sequences along the tooth crown.

## Materials and methods

### The sites

The faunal remains selected for this study derive from four settlements with contemporary and well-dated occupations during the 3rd century BC. These sites are located in distinct ecological and cultural areas in the north-east of the Iberian Peninsula (Fig. 1). Mas Castellar de Pontós is situated on a fertile plain within the northern pre-coastal Catalan region, falling under the territory of Indigeti. Ascending to higher altitudes is the Ceretan settlement of Tossal de Baltarga, located on the Cerdanya plain, in the Pyrenees. Further inland, the Pre-Pyrenees area hosts the settlement of Sant Esteve d’Olius, settled on the fertile Lacetan agricultural plain. Lastly, near the central Mediterranean coast, the Laietan site of Turó de la Rovira extends over the Barcelona plain. Agriculture, and specifically crop cereal production, has traditionally been considered the main economic activity in all four settlements. Indeed, the management, production, storage, and distribution of agricultural surpluses have been documented (Pons et al. 2010; Asensio et al. 2008; Morera 2017). Three of the sites, excluding Tossal de Baltarga, have revealed archaeological evidence of silo fields. However, the Ceretan settlement has preserved, in one of its buildings, large ceramic containers and concentrations of wheat grain, suggesting a probable storage function (Olesti et al. 2018). Animal husbandry, mostly focused on caprines, constituted another key activity within the subsistence economy of the four settlements. Indeed, sheep are the most represented species in all the studied sites, followed by goats, cattle, and pigs (Colominas 2013a, b, 2019; Colominas et al. 2023). The presence of wild taxa, either hunted and consumed or present as commensal species, is minor.

Mas Castellar de Pontós is located in the Empordà region (Girona), near the Mediterranean coast, at 154 m a.s.l. The site is situated approximately 17 km equidistant from the Greek colonies of Rhode and Emporion, on a promontory between two watercourses. Its favourable geographical position made it a strategic communication hub between the coast and the inland regions, as well as between the two Greek colonies and the indigenous inhabitants (Pons et al. 2010). Human occupation of the settlement covers a wide chronological frame, spanning from the late 7th century B.C. to the early 2nd century B.C. (Adroher and Pons 2002; Pons et al. 2010). The phase here investigated extends between 250 BC and 180 B.C., when the site hosted a specialised agricultural settlement associated with an extensive silo field (Pons et al. 2010). This rural settlement was under the governance of a rural aristocracy and played a crucial role as a commercial enclave for cereal production, as well as a reserve centre for the Emporion area (Bouso et al. 2000; Fuertes et al. 2002). The anthracological analyses carried out at the Mas Castellar de Pontós site indicate the existence of mixed holm oak and sessile oak woodlands (*Quercus ilex* and *Quercus petraea*) in the area around the rural establishment, with the presence in addition of juniper (*Juniperus* sp.) and silver fir (*Abies alba*). Moreover, an ecosystem of riparian vegetation (*Alnus* and *Ulmus*) is attested, characteristic of the marshy Empordà plain, rich in ponds and wetlands (Ros and Piqué 2002). Carpological data provide evidence of a great variety of crops, both winter varieties such as wheat and emmer (*Triticum dicoccum*), and spring types such as millet, foxtail millet (*Setaria italica*), and oats (*Avena sativa*). Barley, which is highly represented, can be sown in both the spring and winter seasons. In the earlier phases of the settlement, its association with foxtail millet suggests it was managed as a spring cereal (Bouso et al. 2000; Canal 2002). Barley and millet are predominant, with the latter being the only C_4_ plant, along with foxtail millet, present in the settlement. These cereal crops are combined, in minor quantities, with the cultivation of legumes (pea, lentil), which are important not only for nitrogen fixation in the soil, maintaining a high level of fertility, but also for their high protein content. Among fruits, vines and, to a lesser extent, olive trees are present (Canal 2000, 2002). Cereal production probably involved diverse strategies for intensive crop management, including both rotation systems (biennial and triennial) and the joint sowing of different species, such as foxtail millet and barley (Alonso 2000; Canal 2000). The recovered fauna remains indicate a predominance of domestic species (99% of recorded remains). Among these, caprines are the most represented group (51%), with sheep predominating, followed by cattle and pigs. Hunting was an occasional activity with minimal impact on the diet (Colominas 2013a). However, the few remains of deer, wild boar, and rabbit confirm the proximity of wooded areas mixed with pastures. Regarding sheep husbandry, the prevailing presence of adult and old individuals (Colominas 2008; Colominas 2013a) alongside a bi-modulated reproduction pattern, with births occurring from winter to late spring and in autumn (Messana et al. 2023a), suggest the exploitation of secondary products (i.e., milk and wool) throughout the year.

Tossal de Baltarga (Bellver de Cerdanya, 1166 m a.s.l.) is situated on the top of a hill in the eastern Catalan Pyrenees. The main purpose of the settlement was to exercise territorial control over the communication routes, encompassing both land and the river Segre, that crossed the Cerdanya plain (Colominas et al. 2023). The site has revealed three different phases of occupation spanning from the late Bronze Age to the Republican Roman period (Morera 2017). During the Iron Age phase (4th − 3rd century B.C.) Tossal de Baltarga must have played a relevant social and economic role within the Ceretan population. Indeed, several residential and working buildings linked to a single production unit are attested (Morera 2017; Colominas et al. 2023). Towards the end of the 3rd century BC, the settlement was violently destroyed by fire (Morera 2017). The majority of the archaeobotanical and faunal remains recovered come from one of the buildings (G). Here, charcoal remains of pine (*Pinus sylvestris*/*nigra*), the main taxon, along with silver fir and rose (Rosaceae/Maloideae) were collected (Colominas et al. 2023). The carpological remains, although limited, attest to the presence of barley, wheat, millet, and einkorn (*Triticum monococcum*). However, the highest number of macrobotanical remains corresponds to wild plants (Colominas et al. 2023). These data are supported by the analysis of phytoliths, the majority of which are derived from monocotyledonous plants, particularly grasses and weeds of the Pooideae subfamily (Colominas et al. 2023). The Iberian period witnessed an increase in agricultural production in the Cerdanya plain, favoured by a generally dry climate complemented by abundant water sources (Morera 2017; Olesti and Mercadal 2017). Consequently, during the 3rd century B.C., Cerdanya likely appeared as a plain characterised by an agrarian landscape bordered by forest vegetation essentially consisting of coniferous forests (pine and silver fir), which occupied the mountain area. Building G provided the remains of four sheep, a goat, and, in a separate space, a horse. Consequently, partial or total controlled stabling of livestock would have taken place at Tossal de Baltarga (Morera 2017). All individuals were sub-adults or adults at the time of death caused by the fire. The mortality profiles thus suggest an interest in the products derived from the animals during their lifetimes rather than exclusively for their meat (Colominas et al. 2023). The sequential stable oxygen isotope analyses performed on the molars of three of the sheep show that births at Tossal de Baltarga occurred between late winter and spring, over a relatively narrow timeframe (Messana et al. 2023a). It has been suggested that these births, concentrated in time, were the result of human manipulation to minimise the labour of caring for pregnant females and newborns and ensuring their survival (Messana et al. 2023a). Remains of cattle and pigs were recovered in other buildings, indicating their presence and exploitation within the settlement (Olesti et al. 2024).

The Sant Esteve d’Olius (Solsonès, 664 m a.s.l.) site was a small village located in the fertile Lacetan plain. Situated in the Pre-Pyrenees area, inland in the Catalan region, the settlement was placed on the highest point of a small promontory. The area was bordered by the river Cardener, the principal communication way to the coast (Asensio et al. 2003, 2008). The site was occupied during Medieval and Iron Age times. During the Iron Age phase (3rd c. BC), it emerged as a fortified settlement specialised in storing and managing cereal surpluses, with silo fields and residential buildings (Asensio et al. 2003; Chorén and Calduch 2006; López 2009). The silo field areas around the site outnumber and exceed the designated residential areas, suggesting that agricultural activities were pivotal and must have been a pole of attraction for the other surrounding communities. The expansive fertile plain irrigated by the river Cardener probably played a crucial role in the development of these agricultural activities (Asensio et al. 2008). Although these types of strategic nuclei specialised in crop cereal storage are well known along the Iberian Mediterranean coastal zones, they are exceptional in inland locations, as in the case of Sant Esteve d’Olius (López 2009). The archaeobotanical data from the silos revealed a significant production of cereal crops, especially the winter varieties: barley and, in less proportion, wheat and emmer; as well as some scarce evidence of leguminous plants, such as peas and lentils (López 2009). Among wild plants, species of the Rubiaceae family are the most frequently attested, including catchweed bedstraw or stickwilly (*Galium aparine*, *Galium* sp.), as well as woodruff (*Asperula* sp.) and white bedstraw (*Galium* cf. *mollugo*) in minor quantities. Weedy grasses associated with winter cereals, such as oats (*Avena* sp.), darnel or ryegrass (*Lolium* cf. *temulentum* and *Lolium* sp.) and timothy (*Phelum* sp.) are also present, in addition to other pluvial weeds like *Lithospermum* sp. Furthermore, the archaeological record includes evidence of collected fruits, such as sloe berries (*Prunus spinosa*) and an acorn (*Quercus* sp.; López 2009). The zooarchaeological analysis reveals a predominance of domestic mammal remains. Caprine are prevalent, with sheep more frequent than goats, followed by pigs and cattle (Colominas 2013b). Hunting was a secondary activity with limited impact on the human diet. The presence of wildlife remains such as deer, badger, Leporidae, fox, beech marten, amphibians, and fish confirms the proximity of watercourses and a wetland environment and attests to the presence of wooded areas near the settlement. The mortality profiles of sheep indicate the exploitation of meat and, secondarily, wool and milk (Colominas 2013b). The lambing period, occurring between late winter and early summer, was prolonged compared to the natural one, indicating anthropogenic intervention (Messana et al. 2023a). Furthermore, at Sant Esteve d’Olius the aggregation to the flock is attested for at least one individual with external provenance (and possibly a second one; Messana et al. 2023b).

The Laietan fortified settlement of Turó de la Rovira (mid-3rd century BC – late 3rd /early 2nd century BC) is located in the Barcelona plain, at 262 m. a.s.l., on the top of a small promontory near the Mediterranean coast (Colominas i Roca 1954; Giner 2018). The settlement has been interpreted as a second-order village of the Laietan territory during the Iron Age, occupying a dominant position over the neighbouring minor centres (Giner 2018). Archaeological research has revealed a set of residential buildings on the southern slope and two silo field areas concentrated on the two sides of the village. The settlement was also delimited by the construction of a wall (Giner 2018). Anthracological studies attested to the dominance of open-landscape areas around the site, with abundant remains of strawberry tree (*Arbutus unedo*), holm oak/kermes oak (*Quercus ilex*/*coccifera*), and heather (*Erica* sp.). Deforestation was intense, probably due to the presence of cultivated fields all around (Riera et al. 2020). Carpological data show agriculture with a predominance of wheat and barley, and, in minor quantities, legumes, grapes, and figs. Interestingly, the study also documents the presence, albeit testimonial, of millet (Riera et al. 2020). The majority of the faunal remains come from silos with similar chronology and whose content of archaeocarpological remains is homogeneous (Riera et al. 2020). Caprines are the most attested taxon, with sheep slightly more present than goats. They are followed by pigs and cattle and, in minor numbers, equids, dogs, and Leporidae. Among sheep, adults predominate, indicating their exploitation for wool and meat (Colominas 2019). Both the duration and lambing period, with births occurring between spring and summer, were the result of anthropogenic scheduling aimed at extending the natural birth cycle (Messana et al. 2023a). Finally, a type of short seasonal mobility along a low altitudinal gradient is attested for part of the flock (Messana et al. 2023b). Therefore, it seems possible that there was a differentiated management of the flock within the settlement.

### Materials

#### Bone collagen samples

In this study, a total of 43 sheep (*Ovis aries*, Linnaeus, 1758) remains from the four studied sites were selected for stable carbon and nitrogen isotope analyses. In addition to sheep, a range of other archaeological herbivorous and omnivorous taxa (goat, cattle, pigs, dogs, horses, rabbit, deer, badger, and chicken; *n* = 153), both domestic and wild, were also measured to establish the local δ^13^C and δ^15^N isotopic baseline. The wild species provide information about the environment surrounding the studied sites. On the other hand, domestic taxa offer valuable insights into livestock management practices in the four settlements and enable meaningful comparisons with sheep management strategies. Information regarding species, anatomical element, and age at death of each specimen (*n* = 196) is reported in Table 1. Only remains certainly belonging to different individuals and having a reliable taxonomic determination were selected for this study, considering both laterality and estimated age at death. Only bones from sub-adult or adult individuals were sampled.

Osteological analyses followed the criteria elaborated by Zeder and Pilaar (2010) for teeth and by Boessneck (1969) and Zeder and Lapham (2010) for the post-cranial skeleton were applied to distinguish between goat and sheep. The discrimination between wild boar and domesticated pig was based on the dimensions of anatomical elements and the osteological reference collections of the Institut Català de Paleoecologia Humana i Evolució Social (IPHES-CERCA). Age at death was estimated through the observation of the epiphyseal fusion of long bones, following the methodologies proposed by Barone (1999), and of tooth eruption (Hillson 2005) and wear stage, as described by Payne (1973).

#### Tooth enamel samples

Sequential carbon and oxygen isotope analyses were conducted on 31 lower molars (18 second molars and 13 third molars) belonging to 22 sheep. For nine of these individuals, both M_2_ and M_3_ were sampled. All the selected molars exhibit completely or almost fully formed crowns and early wear stages. The same molars have been analysed in two previous studies aimed at investigating the reproduction patterns and mobility strategies adopted by Iberian populations during the Middle/Late Iron Age (Messana et al. 2023a, b). Table 2 provides descriptive information (side, wear stage, and estimated age of death) about the teeth selected for this study.

**Table 1:** List of animal bone specimens (ID) sampled for bulk collagen analysis. Element and age category of sampled specimens, extraction yield (mg collagen/g bone), collagen carbon (δ^13^C) and nitrogen (δ^15^N) isotope compositions, collagen carbon (%C) and nitrogen (%N) contents, and collagen carbon:nitrogen atomic ratio (C:N)

Sample ID	Taxa	Element	Age Category	Yield (mg/g)	δ^13^C	%C	δ^15^*N*	%*N*	C: *N*
MC OVAR 11,144	*Ovis aries*	Metapod	Adult	8.89	−20.94	43.38	5.80	15.44	3.3
MC OVAR 11,146	*Ovis aries*	Metatarsus	Adult	13.77	−19.64	42.15	5.49	15.70	3.1
MC OVAR 11,159	*Ovis aries*	Calcaneus	Adult	12.59	−19.95	35.70	5.65	12.89	3.2
MC OVAR 11,216	*Ovis aries*	Astragalus	Adult	8.41	−21.42	41.49	6.71	15.03	3.2
MC OVAR 11,217	*Ovis aries*	Tibia	Adult	10.77	−19.40	41.68	5.40	15.19	3.2
MC OVAR 20,129	*Ovis aries*	Phalanx I	Adult	15.81	−21.03	33.80	4.85	13.29	3.0
MC OVAR 20,158	*Ovis aries*	Tibia	Adult	6.88	−20.29	43.51	4.85	15.38	3.3
MC OVAR 20,160	*Ovis aries*	Mandible	Adult	7.19	−19.91	38.65	8.24	14.19	3.2
MC OVAR 20,165	*Ovis aries*	Mandible	Adult	14.66	−19.75	38.96	5.53	15.08	3.0
MC OVAR 20,168	*Ovis aries*	Humerus	Adult	14.40	−19.80	46.84	3.88	16.83	3.2
MC OVAR 100,031	*Ovis aries*	Tibia	Adult	10.38	−20.10	40.71	6.70	14.84	3.2
BTB OVAR 3249.1	*Ovis aries*	Mandible	Adult	7.65	−20.27	36.58	5.61	13.70	3.1
BTB OVAR 3249.3	*Ovis aries*	Radius	Adult	7.09	−20.74	36.75	2.98	14.21	3.0
BTB OVAR 3249.4	*Ovis aries*	Radius	Adult	6.86	−20.77	35.03	5.34	13.29	3.1
BTB OVAR 3270.1	*Ovis aries*	Mandible	Adult	6.22	−19.76	37.41	4.89	13.31	3.3
BTB OVAR 3271.1	*Ovis aries*	Mandible	Subadult	6.75	−19.99	38.09	5.44	14.13	3.1
O OVAR 174	*Ovis aries*	Mandible	Adult	4.93	−20.65	38.35	5.19	13.93	3.2
O OVAR 227	*Ovis aries*	Tibia	Adult	2.92	−20.72	39.64	3.78	14.09	3.3
O OVAR 318	*Ovis aries*	Mandible	Adult	3.42	−20.01	36.65	6.54	13.58	3.1
O OVAR 339	*Ovis aries*	Tibia	Adult	11.59	−20.16	42.22	4.87	15.86	3.1
O OVAR 348	*Ovis aries*	Mandible	Adult	16.65	−20.43	44.87	4.32	16.14	3.2
O OVAR 361	*Ovis aries*	Humerus	Adult	29.82	−20.59	42.04	5.40	15.29	3.2
O OVAR 379	*Ovis aries*	Humerus	Adult	2.07	−20.52	31.71	4.52	12.38	3.0
O OVAR 385	*Ovis aries*	Mandible	Adult	6.29	−20.14	36.70	3.64	13.42	3.2
O OVAR 387	*Ovis aries*	Metacarpus	Adult	4.56	−20.08	34.42	4.29	12.85	3.1
O OVAR 397	*Ovis aries*	Metatarsus	Adult	7.01	−20.24	40.38	7.06	13.57	3.5
O OVAR 405	*Ovis aries*	Mandible	Adult	6.29	−20.27	35.62	4.69	12.39	3.4
O OVAR 438	*Ovis aries*	Radius	Adult	3.35	−20.88	32.76	3.04	13.14	2.9
O OVAR 452	*Ovis aries*	Metacarpus	Adult	13.45	−20.34	44.85	6.50	16.78	3.1
O OVAR 478	*Ovis aries*	Mandible	Adult	4.12	−19.44	32.20	5.28	12.73	3.0
O OVAR 484	*Ovis aries*	Mandible	Adult	14.26	−18.70	43.45	8.21	15.49	3.3
O OVAR 496	*Ovis aries*	Metatarsus	Adult	15.99	−20.85	33.71	6.77	12.04	3.3
O OVAR 500	*Ovis aries*	Radius	Adult	9.95	−22.11	40.29	4.49	14.25	3.3
TR OVAR 415.1	*Ovis aries*	Mandible	Adult	1.08	−19.70	32.97	4.73	13.26	2.9
TR OVAR 415.2	*Ovis aries*	Mandible	Adult	2.94	−20.25	35.20	7.76	11.96	3.4
TR OVAR 439	*Ovis aries*	Tibia	Adult	2.22	−20.54	36.80	4.63	13.93	3.1
TR OVAR 465	*Ovis aries*	Mandible	Adult	12.39	−20.15	43.15	6.58	16.04	3.1
TR OVAR 707	*Ovis aries*	Tibia	Adult	10.35	−20.07	41.48	4.59	14.69	3.3
TR OVAR 725	*Ovis aries*	Mandible	Adult	11.82	−20.58	42.22	5.09	15.75	3.1
TR OVAR 763	*Ovis aries*	Mandible	Adult	5.54	−19.72	38.95	8.21	13.24	3.4
TR OVAR 819	*Ovis aries*	Radius	Adult	10.92	−17.91	38.23	6.86	13.06	3.4
TR OVAR 831	*Ovis aries*	Mandible	Adult	2.49	−18.76	34.70	7.46	13.37	3.0
TR OVAR 1035	*Ovis aries*	Metatarsus	Adult	5.82	−20.31	32.20	5.38	11.22	3.3
MC CAHI 11,144	*Capra hircus*	Mandible	Adult	10.28	−20.16	38.71	5.05	14.75	3.1
MC CAHI 20,160	*Capra hircus*	Mandible	Adult	16.50	−20.50	40.97	4.57	14.71	3.2
MC CAHI 20,162	*Capra hircus*	Humerus	Adult	14.11	−19.97	42.53	5.04	15.08	3.3
MC CAHI 100,030	*Capra hircus*	Tibia	Adult	4.51	−20.40	29.30	3.60	10.73	3.2
BTB CAHI 3250.1	*Capra hircus*	Mandible	Subadult	10.86	−19.74	37.50	6.27	13.44	3.3
O CAHI 181	*Capra hircus*	Metacarpus	Adult	8.62	−20.06	39.61	4.93	14.21	3.3
O CAHI 186	*Capra hircus*	Mandible	Adult	4.11	−20.28	38.01	4.36	12.80	3.5
O CAHI 294	*Capra hircus*	Tibia	Adult	15.72	−19.91	44.05	5.81	15.54	3.3
O CAHI 297	*Capra hircus*	Phalanx I	Adult	19.05	−20.01	23.22	4.65	7.59	3.6
O CAHI 309	*Capra hircus*	Phalanx I	Adult	10.88	−20.42	42.67	3.34	15.63	3.2
O CAHI 403	*Capra hircus*	Phalanx I	Adult	17.65	−19.78	43.03	3.64	15.95	3.1
O CAHI 423	*Capra hircus*	Phalanx I	Adult	17.08	−20.53	41.25	3.31	15.02	3.2
O CAHI 496	*Capra hircus*	Humerus	Adult	7.24	−19.43	30.67	5.32	10.68	3.4
TR CAHI 707	*Capra hircus*	Mandible	Adult	4.49	−20.59	31.73	7.54	12.01	3.1
TR CAHI 719	*Capra hircus*	Mandible	Adult	5.17	−19.97	35.15	3.14	13.08	3.1
TR CAHI 726	*Capra hircus*	Phalanx I	Adult	15.31	−19.51	39.03	4.29	15.90	2.9
TR CAHI 729	*Capra hircus*	Humerus	Adult	16.50	−18.49	43.05	4.33	15.93	3.2
TR CAHI 739	*Capra hircus*	Mandible	Adult	10.69	−20.62	44.00	8.48	15.45	3.3
TR CAHI 748	*Capra hircus*	Phalanx I	Adult	18.33	−20.19	34.14	3.24	13.82	2.9
TR CAHI 805	*Capra hircus*	Mandible	Adult	5.70	−20.19	40.26	2.20	15.06	3.1
TR CAHI 821	*Capra hircus*	Phalanx I	Adult	15.39	−19.73	43.71	4.16	15.58	3.3
TR CAHI 10,004	*Capra hircus*	Astragalus	Adult	101.36	−20.31	35.87	4.84	11.97	3.5
BTB O/C 3051	*Ovis vel Capra*	Humerus	Adult	9.39	−19.72	42.13	7.20	15.00	3.3
BTB O/C 3073	*Ovis vel Capra*	Tibia	Adult	6.04	−20.01	40.43	7.92	15.04	3.1
BTB O/C 3099	*Ovis vel Capra*	Tibia	Adult	16.02	−20.24	41.65	5.08	16.03	3.0
BTB O/C 3159	*Ovis vel Capra*	Ulna	Adult	15.72	−20.74	44.44	4.53	16.49	3.1
MC BOTA 11,125	*Bos taurus*	Mandible	Adult	9.51	−18.86	38.35	5.84	14.22	3.1
MC BOTA 11,145	*Bos taurus*	Metacarpus	Adult	7.75	−20.69	41.31	5.36	15.02	3.2
MC BOTA 11,150	*Bos taurus*	Mandible	Adult	11.61	−18.42	41.18	5.37	15.26	3.1
MC BOTA 20,162	*Bos taurus*	Tibia	Adult	6.70	−19.96	44.18	6.48	16.76	3.1
MC BOTA 20,168	*Bos taurus*	Radius	Adult	6.00	−19.24	44.43	5.20	15.59	3.3
MC BOTA 100,030	*Bos taurus*	Phalanx I	Adult	8.15	−18.30	33.72	5.80	12.47	3.2
BTB BOTA 3031	*Bos taurus*	Mandible	Adult	17.12	−20.43	40.60	3.40	15.16	3.1
BTB BOTA 3051	*Bos taurus*	Mandible	Adult	9.29	−20.74	34.80	5.55	13.00	3.1
BTB BOTA 3064	*Bos taurus*	Phalanx II	Adult	14.09	−20.93	40.61	7.57	16.10	2.9
O BOTA 142	*Bos taurus*	Tibia	Adult	11.76	−20.19	41.92	4.72	15.47	3.2
O BOTA 181	*Bos taurus*	Tibia	Adult	10.59	−21.20	41.47	3.31	14.72	3.3
O BOTA 195	*Bos taurus*	Metacarpus	Adult	6.62	−20.95	39.60	4.70	14.62	3.2
O BOTA 205	*Bos taurus*	Radius	Adult	4.19	−20.71	31.82	4.65	11.21	3.3
O BOTA 318	*Bos taurus*	Humerus	Adult	12.41	−20.34	43.84	5.79	15.10	3.4
O BOTA 320	*Bos taurus*	Humerus	Adult	1.86	−20.42	32.58	4.90	11.95	3.2
O BOTA 329	*Bos taurus*	Radius	Adult	16.43	−20.29	35.78	3.72	13.18	3.2
O BOTA 349	*Bos taurus*	Ulna	Adult	7.16	−20.70	36.98	3.46	13.62	3.2
O BOTA 381	*Bos taurus*	Tibia	Adult	10.47	−20.56	42.71	2.81	15.55	3.2
O BOTA 387	*Bos taurus*	Tibia	Adult	20.83	−22.64	41.41	4.69	15.03	3.2
O BOTA 394	*Bos taurus*	Tibia	Adult	10.90	−20.45	41.12	5.98	15.02	3.2
O BOTA 397	*Bos taurus*	Metatarsus	Adult	3.72	−20.28	28.30	6.92	9.23	3.6
O BOTA 399	*Bos taurus*	Radius	Adult	17.77	−21.32	42.52	4.04	15.25	3.3
O BOTA 423	*Bos taurus*	Humerus	Adult	16.51	−20.14	40.22	4.52	14.35	3.3
O BOTA 448	*Bos taurus*	Humerus	Adult	14.06	−21.78	36.92	1.92	12.93	3.3
O BOTA 482	*Bos taurus*	Metatarsus	Adult	10.42	−20.14	43.32	6.98	15.18	3.3
TR BOTA 412	*Bos taurus*	Femur	Adult	4.60	−21.14	37.45	4.92	14.25	3.1
TR BOTA 415	*Bos taurus*	Phalanx I	Adult	38.43	−20.63	35.34	3.83	13.50	3.1
TR BOTA 439	*Bos taurus*	Metatarsus	Adult	18.98	−21.43	46.76	4.22	17.76	3.1
TR BOTA 707	*Bos taurus*	Metacarpus	Adult	11.17	−20.52	42.43	3.60	16.29	3.0
TR BOTA 715	*Bos taurus*	Metatarsus	Adult	5.66	−19.40	39.03	4.80	15.01	3.0
TR BOTA 719	*Bos taurus*	Mandible	Adult	10.18	−19.47	41.24	6.90	15.55	3.1
TR BOTA 725	*Bos taurus*	Ulna	Adult	11.91	−20.38	40.20	6.70	15.08	3.1
TR BOTA 729	*Bos taurus*	Tibia	Adult	5.23	−18.84	38.72	7.12	14.09	3.2
TR BOTA 739	*Bos taurus*	Tibia	Adult	11.36	−21.00	44.06	2.08	16.47	3.1
TR BOTA 1035	*Bos taurus*	Metacarpus	Adult	4.55	−20.19	34.97	6.22	13.25	3.1
MC SUDO 11,218	*Sus domesticus*	Cranium	Adult	7.15	−18.62	38.31	6.92	13.86	3.2
MC SUDO 20,158	*Sus domesticus*	Mandible	Adult	17.95	−20.39	36.24	5.40	14.02	3.0
MC SUDO 20,168	*Sus domesticus*	Mandible	Adult	14.84	−19.59	43.23	5.51	16.59	3.0
MC SUDO 100,033	*Sus domesticus*	Ulna	Adult	11.28	−20.50	42.20	4.30	15.34	3.2
BTB SUDO 3029	*Sus domesticus*	Mandible	Adult	6.91	−20.18	40.25	5.61	15.29	3.1
BTB SUDO 3051	*Sus domesticus*	Mandible	Adult	5.96	−20.14	40.94	8.43	15.28	3.1
O SUDO 186	*Sus domesticus*	Mandible	Adult	5.85	−19.80	37.01	6.32	12.23	3.5
O SUDO 205	*Sus domesticus*	Mandible	Adult	3.86	−20.25	35.22	4.42	12.58	3.3
O SUDO 235	*Sus domesticus*	Radius	Adult	6.46	−20.27	38.24	4.20	13.59	3.3
O SUDO 245	*Sus domesticus*	Mandible	Adult	8.52	−20.45	40.88	7.83	14.14	3.4
O SUDO 309	*Sus domesticus*	Tibia	Adult	5.92	−20.42	37.63	10.18	12.57	3.5
O SUDO 311	*Sus domesticus*	Mandible	Adult	1.28	−20.32	31.85	4.00	12.51	3.0
O SUDO 318	*Sus domesticus*	Tibia	Adult	14.94	−20.32	43.20	6.24	14.67	3.4
O SUDO 329	*Sus domesticus*	Maxilla	Adult	9.24	−19.94	39.31	8.50	14.06	3.3
O SUDO 371	*Sus domesticus*	Calcaneus	Adult	6.36	−20.61	40.90	7.03	15.02	3.2
O SUDO 385.1	*Sus domesticus*	Mandible	Adult	4.89	−20.96	38.12	7.69	13.67	3.3
O SUDO 385.2	*Sus domesticus*	Mandible	Subadult	6.21	−20.55	37.96	5.51	14.23	3.1
O SUDO 397	*Sus domesticus*	Mandible	Adult	14.48	−20.80	43.49	4.08	15.91	3.2
O SUDO 399	*Sus domesticus*	Maxilla	Adult	37.35	−20.03	36.71	4.33	13.81	3.1
O SUDO 427	*Sus domesticus*	Calcaneus	Adult	21.19	−20.78	34.97	5.72	13.26	3.1
O SUDO 482	*Sus domesticus*	Mandible	Adult	3.52	−20.34	38.22	7.12	14.87	3.0
O SUDO 486	*Sus domesticus*	Ulna	Adult	6.85	−20.07	38.18	5.56	14.13	3.2
TR SUDO 415	*Sus domesticus*	Humerus	Adult	3.52	−20.88	37.74	6.79	14.15	3.1
TR SUDO 465	*Sus domesticus*	Metapod	Adult	14.19	−20.39	42.01	6.16	15.60	3.1
TR SUDO 707	*Sus domesticus*	Humerus	Adult	6.20	−19.56	37.55	4.80	14.14	3.1
TR SUDO 725	*Sus domesticus*	Mandible	Adult	8.50	−20.32	41.28	6.19	14.60	3.3
TR SUDO 739	*Sus domesticus*	Mandible	Adult	4.90	−20.27	27.97	5.68	9.36	3.5
TR SUDO 748	*Sus domesticus*	Metapod	Adult	11.32	−20.30	41.62	6.20	15.39	3.2
TR SUDO 765	*Sus domesticus*	Metapod	Adult	12.70	−20.55	41.94	4.67	15.30	3.2
TR SUDO 766	*Sus domesticus*	Scapula	Adult	8.00	−20.50	40.54	7.09	14.22	3.3
TR SUDO 821	*Sus domesticus*	Metapod	Adult	13.10	−20.80	41.68	5.14	15.98	3.0
TR SUDO 1065	*Sus domesticus*	Metapod	Adult	5.46	−20.76	41.07	4.36	15.65	3.1
MC EQCA 11,123	*Equus caballus*	Metatarsus	Adult	5.06	−18.94	26.69	5.33	9.58	3.2
MC EQCA 11,159	*Equus caballus*	Phalanx I	Adult	5.92	−21.61	37.70	5.87	14.29	3.1
MC EQCA 20,160	*Equus caballus*	Phalanx II	Adult	4.68	−19.63	30.26	4.96	11.16	3.2
MC EQCA 20,165	*Equus caballus*	Tibia	Adult	10.78	−20.03	40.83	5.76	15.07	3.2
MC EQCA 20,168	*Equus caballus*	Humerus	Adult	11.83	−19.87	44.75	5.13	17.07	3.1
O EQCA 148	*Equus caballus*	Scapula	Adult	12.08	−21.40	30.26	1.82	10.48	3.4
O EQCA 397	*Equus caballus*	Mandible	Adult	2.57	−21.07	33.35	6.97	11.69	3.3
TR EQCA 415	*Equus caballus*	Mandible	Adult	14.11	−20.36	43.34	3.42	16.89	3.0
TR EQCA 439	*Equus caballus*	Pelvis	Adult	14.71	−20.42	42.47	4.32	14.71	3.4
TR EQCA 729	*Equus caballus*	Humerus	Adult	6.82	−21.71	41.15	3.73	15.87	3.0
TR EQCA 805	*Equus caballus*	Femur	Adult	4.00	−19.19	38.70	6.63	14.39	3.1
TR EQCA 1037	*Equus caballus*	Mandible	Adult	3.88	−18.67	37.79	4.33	14.72	3.0
O CEEL 205	*Cervus elaphus*	Phalanx I	Adult	11.58	−20.24	42.05	2.15	16.01	3.1
O CEEL 245	*Cervus elaphus*	Tibia	Adult	11.32	−21.04	42.64	3.27	14.87	3.3
O CEEL 309	*Cervus elaphus*	Metatarsus	Adult	9.45	−21.65	41.18	3.41	14.90	3.2
O CEEL 339	*Cervus elaphus*	Metatarsus	Adult	14.71	−21.07	37.97	4.66	13.18	3.4
O CEEL 387	*Cervus elaphus*	Radius	Adult	12.49	−20.71	43.16	3.49	15.47	3.3
O CEEL 421	*Cervus elaphus*	Phalanx I	Adult	9.43	−21.08	40.36	3.40	14.56	3.2
O CEEL 452	*Cervus elaphus*	Phalanx I	Adult	64.80	−21.73	42.04	3.28	15.47	3.2
MC CAFA 12028.1	*Canis familiaris*	Humerus	Adult	9.16	−19.72	41.60	6.79	15.30	3.2
MC CAFA 12028.2	*Canis familiaris*	Humerus	Adult	9.58	−20.47	38.83	5.61	14.30	3.2
MC CAFA 12028.3	*Canis familiaris*	Ulna	Adult	9.16	−16.97	39.99	5.94	15.16	3.1
MC CAFA 20,165	*Canis familiaris*	Mandible	Adult	17.92	−18.25	42.17	7.27	15.53	3.2
MC CAFA 100,031	*Canis familiaris*	Metacarpus	Adult	4.29	−17.50	33.70	8.40	12.62	3.1
O CAFA 349	*Canis familiaris*	Ulna	Adult	12.52	−19.19	41.71	5.27	15.37	3.2
TR CAFA 415	*Canis familiaris*	Radius	Adult	1.98	−19.98	15.45	8.21	5.38	3.3
TR CAFA 719	*Canis familiaris*	Metapod	Adult	9.27	−18.32	37.00	10.07	12.64	3.4
TR CAFA 726	*Canis familiaris*	Pelvis	Adult	6.67	−20.09	37.83	7.85	14.55	3.0
TR CAFA 766	*Canis familiaris*	Metapod	Adult	15.70	−20.15	40.45	7.15	14.28	3.3
TR CAFA 819	*Canis familiaris*	Metapod	Adult	10.13	−19.66	42.12	8.83	15.62	3.1
O MEME 257	*Meles meles*	Tibia	Adult	7.08	−18.64	41.84	7.14	15.05	3.2
MC AV 11,221	*Milvus milvus*	Humerus	Adult	24.74	−18.20	40.76	7.42	14.67	3.2
MC AV 11,145	Bird	Ulna	Adult	14.46	−21.30	41.71	6.11	16.33	3.0
MC AV 20,158	Bird	Radius	Adult	18.06	−20.81	41.28	6.74	14.40	3.3
O AV 186.1	*Gallus gallus*	Tarsus-Metatarsus	Adult	15.25	−18.92	44.25	7.27	15.59	3.3
O AV 186.2	*Gallus gallus*	Tarsus-Metatarsus	Adult	16.88	−18.45	43.25	7.98	16.25	3.1
O AV 205	*Gallus gallus*	Tibia-Tarsus	Adult	12.18	−19.43	42.43	8.15	14.57	3.4
O AV 361	*Gallus gallus*	Tarsus-Metatarsus	Adult	9.98	−18.88	35.46	8.23	12.95	3.2
O AV 387	*Gallus gallus*	Tibia-Tarsus	Adult	12.07	−19.56	34.52	7.29	12.52	3.2
TR AV 528.1	*Gallus gallus*	Humerus	Adult	9.79	−19.51	42.72	8.59	15.92	3.1
TR AV 528.2	*Gallus gallus*	Humerus	Adult	15.92	−20.03	32.85	9.82	12.24	3.1
TR AV 719	*Gallus gallus*	Tarsus-Metatarsus	Adult	12.42	−19.76	40.83	9.21	15.92	3.0
TR AV 763	*Gallus gallus*	Femur	Adult	13.14	−20.54	45.00	7.44	16.61	3.2
TR AV 764.1	*Gallus gallus*	Tibia-Tarsus	Adult	11.35	−20.27	41.60	10.27	14.36	3.4
TR AV 764.2	*Gallus gallus*	Tibia-Tarsus	Adult	14.56	−20.58	47.87	7.26	17.20	3.2
TR AV 765	*Gallus gallus*	Tibia-Tarsus	Adult	12.10	−20.73	36.52	7.64	13.19	3.2
MC ORCU 20,122	*Oryctolagus cuniculus*	Humerus	Adult	9.91	−21.54	39.72	3.08	14.07	3.3
MC ORCU 20,158	*Oryctolagus cuniculus*	Pelvis	Adult	13.40	−21.52	43.26	3.76	16.07	3.1
MC ORCU 20,160	*Oryctolagus cuniculus*	Radius	Adult	14.97	−22.05	40.27	5.34	14.77	3.2
MC ORCU 20,162	*Oryctolagus cuniculus*	Radius	Adult	16.53	−21.56	41.24	3.45	15.50	3.1
MC ORCU 20165.1	*Oryctolagus cuniculus*	Ulna	Adult	13.68	−21.14	41.88	3.95	15.41	3.2
MC ORCU 20165.2	*Oryctolagus cuniculus*	Ulna	Adult	11.66	−21.15	42.37	3.76	15.84	3.1
O ORCU 381	*Oryctolagus cuniculus*	Tibia	Adult	10.66	−21.43	35.35	9.42	12.31	3.4
O ORCU 397	*Oryctolagus cuniculus*	Tibia	Adult	13.45	−21.97	42.04	1.99	15.11	3.2
O ORCU 459	*Oryctolagus cuniculus*	Tibia	Adult	13.34	−22.94	34.31	4.38	12.70	3.2
O ORCU 486.1	*Oryctolagus cuniculus*	Tibia	Adult	13.06	−22.48	40.70	8.18	14.71	3.2
O ORCU 486.2	*Oryctolagus cuniculus*	Tibia	Adult	13.91	−19.65	41.41	5.66	15.75	3.1
TR ORCU 439	*Oryctolagus cuniculus*	Pelvis	Subadult	6.30	−21.61	34.14	6.79	13.38	3.0
TR ORCU 463	*Oryctolagus cuniculus*	Humerus	Adult	10.76	−20.27	40.72	4.31	15.13	3.1
TR ORCU 739	*Oryctolagus cuniculus*	Metapod	Adult	5.97	−21.93	31.30	9.97	11.69	3.1

**Table 2:** Sheep tooth specimens (ID) sampled for the sequential carbon and oxygen analyses in this study: site, side (L, left; R, right), wear stages and estimated age of death according to Payne (1973); na, no analysed

			M_2_		M_3_	
Site	Teeth ID	Side	Wear stage	Age estimation	Wear stage	Age estimation
Mas Castellar de Pontós (MC)	11144	L	F	2–6 years	na	na
	12023	L	E	2–3 years	na	na
	20102	L	E	2–3 years	na	na
	20160	R	E	2–3 years	F	3–4 years
	20165	R	F	3–4 years	F	3–4 years
	11132	R	na	na	G	4–6 years
	11134	L	na	na	G	4–6 years
	11138	L	na	na	G	4–6 years
Tossal de Baltarga (BTB)	3249	R	E	2–3 years	na	na
	3270	R	F	3–4 years	F	3–4 years
	3271	L	D	21–24 months	na	na
	3031	L	na	na	F	3–4 years
Sant Esteve d’Olius (O)	174	L	E	2–3 years	na	na
	205	R	E	2–3 years	E	2–3 years
	318	R	G	4–5 years	G	4–5 years
	348	R	F	4–6 years	na	na
	478	L	E	2–3 years	E	2–3 years
	482	R	F	4–6 years	na	na
	484	R	F	4–6 years	na	na
Turó de la Rovira (TR)	725	R	F	3–4 years	F	3–4 years
	763	L	F	3–4 years	F	3–4 years
	831	L	E	2–3 years	E	2–3 years

### Methods

#### Bone collagen extraction and carbon and nitrogen isotope analyses

Sample preparation and collagen extraction were performed at the Biomarkers Laboratory of the Institut Català de Paleoecologia Humana i Evolució Social (IPHES-CERCA). A small fragment of bone was cut and removed from each specimen using a Dremel diamond rotating wheel. The surface of the bone shards was cleaned by abrasion with a tungsten drill to remove any visible contaminants. Collagen extraction followed the protocol proposed by Longin (1971) and modified by Bocherens et al. (1991). The bone fragments were initially soaked in 0.5 M HCl for demineralisation, rinsed several times with distilled water, and soaked in NaOH (0.125 M) for 20 h to remove potential organic contaminants. After another round of rinsing, the samples were diluted and gelatinised with 0.01 M HCl at 100 °C for 17 h (pH 2). Finally, collagen samples were microfiltered using 5 μm filters to remove any residues and subsequently frozen. Samples were lyophilised at the Institut de Ciència i Tecnologia Ambientals (ICTA-UAB). Gelatin-collagen samples weighing about 300 μm were set into tin capsules and analysed at the Institut de Ciència i Tecnologia Ambientals (ICTA-UAB), using a Thermo Flash 1112 elemental analyser (EA) coupled to a Thermo Delta V Advantage isotope ratio mass spectrometer (IRMS) with a Conflo III interface. The mean analytical precision was verified using the international standard laboratory IAEA 600 (caffeine; theoretical values: δ^13^C = −27.77 ± 0.04‰, δ^15^N = + 1.0 ± 0.2‰) and used for data calibration. The average analytical error was < 0.05‰ (1σ) for δ^13^C and ± 0.13‰ δ^15^N, determined within each run and from repeated measurements of the standard. The isotope composition is reported in δ notation and expressed in per mil (‰). The isotope ratios are reported for carbon as δ^13^C Vienna Pee Dee Belemnite (V-PDB) and for nitrogen as δ^15^N atmospheric nitrogen (AIR): δX(‰) = [(R_sample_/R_standard_) – 1] ×1000, where X stands for ^13^ or ^15^ N and R stands for ^13^C/^12^ or ^15^ N/^14^N. To evaluate the reliability of the isotopic signature of the collagen extracts, several quality criteria were applied: a yield of extraction ≥ 10 mg·g − 1, percentages of C ≥ 30% and *N* ≥ 10%, and a range of atomic C:N ratio between 2.9 and 3.6 (DeNiro 1985; Ambrose 1990; Van Klinken 1999).

To test the statistical significance of the isotopic values between groups, we first evaluate the normality of the data distribution using the Shapiro-Wilk test (*α* = 0.05). Depending on the outcome, we either conduct the one-way ANOVA test for normally distributed data or the Kruskal-Wallis test for non-normally distributed data, using Past software (version 4.11) and setting the significance *p*-value at < 0.05. In case of significant differences between data sets, we apply appropriate post-hoc tests: Tukey’s pairwise test for parametric data and Dunn’s test for non-parametric data.

#### Sequential carbon and oxygen isotope analysis

Before sampling, the surface of the selected teeth was cleaned by abrasion with a tungsten drill. Subsequently, enamel bands were sequentially sampled with a diamond drill on the buccal surface, on the distal lobe of M_2_ and the central lobe of M_3_. Samples were taken at 1–2 mm intervals from the apex to the enamel-root junction (ERJ) of each tooth. The positions of the sample bands were recorded in millimetres along the entire crown, starting from the ERJ. The drilling and the chemical treatment of the samples were performed at the Biomarkers Laboratory of the Institut Català de Paleoecologia Humana i Evolució Social (IPHES-CERCA). A total of 473 enamel powder samples (between 10 and 20 samples per tooth) were collected, weighing between 2.5 and 13.7 mg. Following the protocol described by Balasse et al. (2002) and modified by Tornero et al. (2013), the bioapatite samples were treated to remove exogenous carbonate contamination (4 h in 0.1 M acetic acid [CH3COOH]; 0.1 ml solution/mg sample), rinsed multiple times in distilled water and dried in an oven at 70 °C for 48 h. Once treated, samples were measured using an automated carbonate preparation device (KIEL-III) interfaced with a Finnigan MAT 252 isotope ratio mass spectrometer (IRMS) at the Environmental Isotope Laboratory (Department of Geosciences), University of Arizona (USA), under the scientific supervision of Dr. David Dettman. Powdered samples were reacted with dehydrated phosphoric acid under vacuum at 70 °C. To ensure the accuracy and precision of measurements, NBS-19 and NBS-18 international standards were utilized. The mean analytical precision within each run and from replicate measurements of standards during analysis varies ± 0.1‰ for δ^18^O and ± 0.08‰ for δ^13^C (1σ). The isotope composition is normalised to the Vienna-Pee Dee Belemnite (V-PDB) standard for both carbon and oxygen values.

## Results

### Collagen preservation and δ^13^C and δ^15^N analysis results

The carbon and nitrogen isotopic composition and collagen quality indicators of each individual are reported in Table 1. Collagen was successfully extracted from all samples analysed. The majority of the collagen samples exhibit carbon and nitrogen content values and C:N ratios are in accordance with the quality criteria outlined in the literature (DeNiro 1985; Ambrose 1990; Van Klinken 1999). Collagen yields range from 1.08 to 101.36 mg/g. Only samples with a C:N ratio falling within the ranges of 2.9 to 3.5, wt% C > 30, and wt% *N* > 10 were considered for final interpretation. This led to the exclusion of six individuals: MC CAHI 100030, MC EQCA 11123, O CAHI 297, O BOTA 397, TR SUDO 739, and TR CAFA 415. The mean of the C:N ratios of the 190 samples is 3.2 ± 0.1. The carbon content (in wt% C) exhibits a mean value of 39.4% ± 3.68%, whilst the nitrogen content (in wt% N) displays a mean value of 14.5% ± 1.39%.

**Fig. 2:**
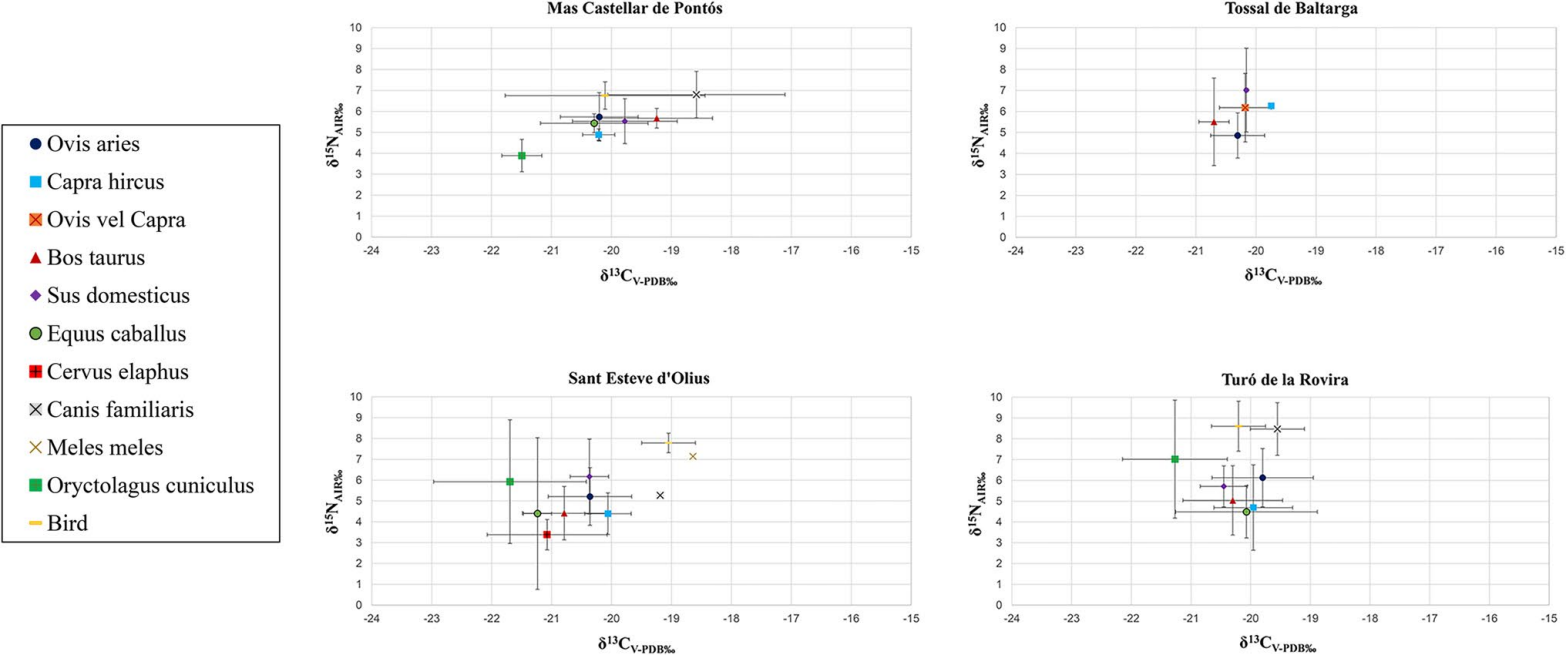
Carbon (δ^13^C_V−PDB‰_) and nitrogen (δ^15^N_AIR‰_) values (mean and sd) of bone collagen samples from the four Iberian Iron Age sites

The mean of the δ^13^C and δ^15^N isotope values of the faunal remains (corresponding to adult specimens) are − 20.3 ± 0.91‰ and 5.6 ± 1.78‰, respectively. All δ^13^C values fall within the expected range for a diet primarily composed of C_3_ plants. Likewise, all δ^15^N values fit within the range for a diet encompassing both terrestrial plant and animal protein sources. The mean and range of δ^13^C and δ^15^N values of the samples analysed for every species at each site are illustrated in Fig. 2. The summary data (number of samples, mean and standard deviation of δ^13^C and δ^15^N values) for the species analysed in this study are reported in Table 3a. Corresponding data for comparison individuals from the literature are provided in Table 3b. The results from the post-hoc tests are included in Table S1.

Collagen samples from sheep from the four studied sites display a δ^13^C mean value of −20.2 ± 0.71‰ and a δ^15^N mean value of 5.5 ± 1.33‰ (*n* = 43). The Kruskal-Wallis test was conducted to compare δ^13^C and δ^15^N values of the sheep samples from the four sites. The results revealed that there are no statistically significant differences in either δ^13^C (Kruskal-Wallis test: *H* = 3.48; *p* = 0.32) and δ^15^N values (Kruskal-Wallis test: *H* = 4.19; *p* = 0.24) between the sites. Comparison with results obtained from four sheep remains dated to the Roman imperial period (II-III AD) and recovered from the necropolis of the Vila de Madrid site (Barcelona) shows no substantial differences in either δ^13^C (Kruskal-Wallis test: *H* = 4.03; *p* = 0.40) and δ^15^N values (Kruskal-Wallis test: *H* = 4.17; *p* = 0.38; Salazar-García et al. 2022).

For *Capra hircus* (*n* = 20), the mean δ^13^C value is −20.0 ± 0.50‰, and the mean δ^15^N value is 4.7 ± 1.51‰. There are no statistically significant differences in δ^13^C (ANOVA test: *F* = 0.27; *p* = 0.77) and δ^15^N values (Kruskal-Wallis test: *H* = 1.48; *p* = 0.48) between the four sites. Comparisons can be made between the δ^13^C and δ^15^N values of the goat samples and those of two individuals from two Catalan sites: Can Roqueta (Barcelona, 1900–16000 cal BC) and Minferri (Lleida, 2100 − 1650 cal BC), both dating to the Early and Middle Bronze Age. The current data indicates that there are no notable differences in the δ^13^C (ANOVA test: *F* = 1.70; *p* = 0.20) and δ^15^N values (Kruskal-Wallis test: *H* = 1.69; *p* = 0.64) between these periods (Grandal-d’Anglade et al. 2019).

**Table 3:** Summary data for the species analysed in this study: number of samples, mean and standard deviation of δ^13^C and δ^15^N values (table 3a); Summary data for comparison individuals from the literature: number of samples, mean and standard deviation of δ^13^C and δ^15^N values (table 3b)

Taxon	Site	*n*	δ^13^C_V−PDB‰_ mean	δ^13^C_V−PDB‰_ sd	δ^15^*N*_AIR‰_ mean	δ^15^*N*_AIR‰_ sd	
*Ovis aries*	MC	11	−20.2	0.65	5.7	1.16	
	BTB	5	−20.3	0.45	4.9	1.08	
	O	17	−20.4	0.70	5.2	1.38	
	TR	10	−19.8	0.85	6.1	1.40	
	All	43	−20.2	0.71	5.5	1.33	
*Capra hircus*	MC	3	−20.2	0.27	4.9	0.27	
	BTB	1	−19.7		6.3		
	O	7	−20.1	0.39	4.4	1.00	
	TR	9	−20	0.66	4.7	2.05	
	All	20	−20	0.50	4.7	1.51	
*Bos taurus*	MC	7	−19.3	0.85	5.9	0.74	
	BTB	3	−20.7	0.25	5.5	2.09	
	O	15	−20.8	0.70	4.4	1.28	
	TR	10	−20.3	0.84	5	1.67	
	All	35	−20.3	0.93	5	1.45	
*Sus domesticus*	MC	4	−19.8	0.87	5.5	1.07	
	BTB	2	−20.2	0.03	7	2.00	
	O	16	−20.4	0.32	6.2	1.80	
	TR	9	−20.5	0.39	5.7	0.99	
	All	31	−20.3	0.47	6	1.51	
*Equus caballus*	MC	4	−20.3	0.90	5.4	0.45	
	O	2	−21.2	0.24	4.4	3.64	
	TR	5	−20.1	1.19	4.5	1.26	
	All	11	−20.4	1.00	4.8	1.50	
*Canis familiaris*	MC	5	−18.6	1.48	6.8	1.11	
	O	1	−19.2		5.3		
	TR	4	−19.6	0.85	8.5	1.27	
	All	10	−19	1.20	7.3	1.51	
*Cervus elaphus*	O	7	−21.1	0.52	3.4	0.73	
*Meles meles*	O	1	−18.6		7.1		
Aves	MC	3	−20.1	1.67	6.8	0.65	
*Gallus gallus*	O	5	−19	0.45	7.8	0.47	
	TR	7	−20.2	0.45	8.6	1.20	
	All	12	−19.7	0.73	8.3	1.02	
Taxon	Site	Chronology	*n*	δ^13^C_V−PDB‰_ mean	δ^13^C_V−PDB‰_ sd	δ^15^*N*_AIR‰_ mean	δ^15^*N*_AIR‰_ sd
*Ovis aries*	Vila de Madrid	II-III AD	4	−19.8	0.91	5.3	1.41
*Capra hircus*	Minferri	E-MBA	1	−18.9		6.5	
	Can Roqueta	E-MBA	1	−19.5		4.2	
*Bos taurus*	Can Roqueta	E-MBA	5	−19.4	0.56	5.7	0.58
	Minferri	E-MBA	6	−19.8	0.45	6.4	1.35
	Can Roqueta	LBA	6	−18.8	1.28	6.2	0.78
	Can Roqueta	EIA	8	−17.8	1.15	7.2	1.28
	Vila de Madrid	II-III AD	7	−21	0.43	3.6	1.55
	Carrer Ample 1	I-IV AD	4	−21.1	0.64	2.8	0.66
*Sus domesticus*	Minferri	E-MBA	2	−20.2	0.14	8.6	0.92
	Can Roqueta	LBA	2	−18.1	0.85	8.7	1.77
	Can Roqueta	EIA	6	−19.5	0.64	8	0.66
	Vila de Madrid	II-III AD	6	−20.6	0.46	4.7	2.21
	Carrer Ample 1	I-IV AD	6	−20	0.34	5.4	2.14
*Equus caballus*	Can Roqueta	LBA	6	−18.9	1.33	4.7	1.15
	Can Roqueta	EIA	28	−19.4	1.45	5.8	0.84
	Can Roqueta	E-MIA/IP	2	−20.4	0.07	6.2	1.06
	Vila de Madrid	II-III AD	8	−21.2	0.43	4.2	0.94
	Carrer Ample 1	I-IV AD	3	−19.8	0.76	4.3	0.78
*Canis familiaris*	Can Roqueta	E-MBA	16	−19.3	0.31	7.7	0.59
	Minferri	E-MBA	7	−19.0	0.27	8.8	0.24
	Can Roqueta	LBA	8	−18.8	0.73	8.1	1.36
	Can Roqueta	LBA/EIA	2	−17.7	0.99	9.3	0.28
	Can Roqueta	EIA	13	−17.9	0.97	8.9	0.70
	Vila de Madrid	II-III AD	15	−18.9	0.34	8.8	1.48
*Cervus elaphus*	Carrer Ample 1	I-IV AD	5	−20.1	0.11	3.1	0.84
*Meles meles*	Can Roqueta	EIA	1	−18.8		7.6	
*Gallus gallus*	Vila de Madrid	II-III AD	4	−19	1.29	8.6	1.24
	Carrer Ample 1	I-IV AD	1	−18.6		10.3	
*Oryctolagus cuniculus*	Vila de Madrid	II-III AD	2	−21.9	1.41	3.6	1.91

For *Bos taurus* (*n* = 35), the mean δ^13^C value is −20.3 ± 0.93‰, and the mean δ^15^N value is 5.0 ± 1.45‰. Among the four sites, a difference can be noted in the diet of individuals from Mas Castellar de Pontós, displaying higher δ^13^C values (mean value − 19.26‰ ± 0.85‰), suggesting a slight integration of C_4_ plants, in contrast to the predominantly C_3_ diet of those from Tossal de Baltarga, Sant Esteve d’Olius, and Turó de la Rovira (Kruskal-Wallis tests: *H* = 10.95; *p* = 0.01; Table S1a). No statistically significant difference is observed in δ^15^N values (ANOVA test: *F* = 2.008; *p* = 0.13). The existing literature allows comparisons with various sites of proximate chronologies: Can Roqueta (different levels dated to E-MBA, LBA, EIA), Minferri (E-MBA), Vila de Madrid (RP), Carrer Ample 1 (RP). There are significant differences in the δ^13^C values between chronologies (Kruskal-Wallis test: *H* = 48.66; *p* = 1.91E-07; Table S1b). The five individuals from the E-MBA levels of Can Roqueta and the six individuals from the LBA levels show a mean δ^13^C value of −19.4 ± 1.20‰ and − 18.8 ± 3.40‰, respectively (Grandal-d’Anglade et al. 2019; Albizuri et al. 2021). These data might suggest the integration of C_4_ plants into their diet, contrasting with the results obtained for cattle from Tossal de Baltarga, Sant Esteve d’Olius, and Turó de la Rovira. Individuals from the LBA levels of Minferri (*n* = 6) exhibit a mean δ^13^C value of −19.8 ± 1.30‰, displaying a different diet in comparison to the cattle from Sant Esteve d’Olius (Grandal-d’Anglade et al. 2019). Indeed, the latter settlement displays the lowest δ^13^C values for cattle remains among the studied sites. For the EIA levels at Can Roqueta, the bone remains of eight cattle were analysed. These exhibit a mean δ^13^C value of −17.8 ± 3.50‰ and display great variability (Albizuri et al. 2021). This suggests that C_4_ plants made an important contribution to their diet, contrary to the dietary patterns observed at the four studied sites. In the Roman period, individuals from Vila de Madrid and Carrer Ample 1 sites display different δ^13^C values in comparison to the cattle from Mas Castellar de Pontós (Rissech et al. 2016; Salazar-García et al. 2022). Concerning δ^15^N values, there are no statistically significant differences between cattle from the four sites studied and those from the E-MBA and LBA levels of Can Roqueta and from the E-MBA of Minferri (Grandal-d’Anglade et al. 2019; Albizuri et al. 2021; Table S1c). On the other hand, statistically significant differences are observed when comparing the eight individuals from the EIA of Can Roqueta and the Roman Period cattle from Vila de Madrid and Carrer Ample 1 (Rissech et al. 2016; Grandal-d’Anglade et al. 2019; Albizuri et al. 2021; Salazar-García et al. 2022; Table S1c).

For *Sus domesticus* (*n* = 31), the mean δ^13^C value is −20.3 ± 0.47‰, and the mean δ^15^N value is 6.0 ± 1.51‰. Pigs from the Iron Age studied sites exhibit a diet primarily based on C_3_ plants. Only the individuals from Mas Castellar de Pontós present enriched δ^13^C values (mean value of −19.78‰ ± 0.87‰), suggesting a potential contribution of C_4_ plants to their diet. Nevertheless, there are no statistically significant differences among pigs from the four sites (ANOVA test: *F* = 2.53; *p* = 0.08). Individuals from Tossal de Baltarga and Sant Esteve d’Olius display higher δ^15^N values compared to those from Mas Castellar de Pontós and Turó de la Rovira, although there are no statistically significant differences (ANOVA test: *F* = 0.59; *p* = 0.63). It is possible to compare the δ^13^C and δ^15^N values of these pigs with those of individuals from Minferri (E-MBA), as well as from both the E-MBA and LBA levels of Can Roqueta, from Vila de Madrid and Carrer Ample 1 (RP). In general, Minferri pigs predominantly consume C_3_ plants (mean δ^13^C values of −20.2 ± 0.20‰) and exhibit higher δ^15^N values (mean of 8.6 ± 1.30‰) compared to the Iron Age individuals, especially those from Mas Castellar de Pontós and Turó de la Rovira (Grandal-d’Anglade et al. 2019). However, there are no statistical differences in δ^13^C values. The two individuals from the LBA phase at Can Roqueta display a mean δ^13^C value of −18.1 ± 1.20‰, suggesting a possible contribution of C_4_ plants to the pigs’ diet. Indeed, their δ^13^C values differ significantly from the ones of the four Iberian sites (Table S1d). The mean δ^15^N value is 8.7 ± 2.50‰, consistent with the average value observed during the E-MBA at Minferri and higher than the mean value of Iberian pigs (Albizuri et al. 2021). During the EIA at Can Roqueta, the pigs were fed mainly on C_3_ plants (mean δ^13^C values of −19.5 ± 1.80‰) and show δ^15^N values in line with previous chronologies, although it showed a slight decrease, with mean δ^15^N values of 8.0 ± 1.70‰ (Albizuri et al. 2021). Pigs dating to the Roman period from Vila de Madrid (*n* = 6) and Carrer Ample 1 (*n* = 6) were fed on C_3_ plants (mean δ^13^C values of −20.6 ± 1.30‰ and − 20.0 ± 0.90‰, respectively). Individuals from Vila de Madrid exhibit a mean δ^15^N value of 4.7 ± 6.00‰, while those from Carrer Ample 1 display a mean δ^15^N value of 5.4 ± 5.70‰ (Rissech et al. 2016; Salazar-García et al. 2022). These values suggest a diet with a lower protein content than that observed for E-MBA, LBA, and EIA and generally in line with the diet of Iron Age pigs. Nevertheless, the difference in δ^15^N values between the pigs from Vila de Madrid and the ones from the EIA of Can Roqueta is the sole statistically significant (Table S1e).

When it comes to *Equus caballus* (*n* = 11), the mean δ^13^C value is −20.4 ± 1.00‰, and the mean δ^15^N value is 4.8 ± 1.50‰. There are no statistically significant differences in δ^13^C (ANOVA test: *F* = 0.97; *p* = 0.42) and δ^15^N values (ANOVA test: *F* = 0.48; *p* = 0.64) between the Iberian sites. The comparison with six horses from the LBA phase of Can Roqueta and 28 from the EIA phase does not show substantial differences in feeding habits, except for a slightly increased consumption of C_4_ plants in the forage in comparison with the horses from Sant Esteve d’Olius (a mean δ^13^C value of −18.9 ± 2.90‰ and − 19.4 ± 4.80‰, for the LBA and EIA phases respectively; Grandal-d’Anglade et al. 2021; Table S1f). Comparison with two individuals from the E-MIA/IP levels shows no differences in the horses’ diet with respect to individuals from the Iron Age period (Table S1f). Indeed, the mean δ^13^C value at Can Roqueta is −20.4 ± 0.10‰ (Grandal-d’Anglade et al. 2021). Horses dated to the Roman period from the necropolis of Vila de Madrid show a mean δ^13^C value of −21.2 ± 1.30‰, which is in line with a diet primarily composed of C_3_ plants, similar to the Iron Age period (Salazar-García et al. 2022). The three individuals of the LR from Carrer Ample 1 show a slight integration of C_4_ plants in the diet (mean δ^13^C values of −19.8 ± 1.50‰), although not statistically significant (Table S1f; Grandal-d’Anglade et al. 2021).

For *Canis familiaris* (*n* = 10), the mean δ^13^C value is −19.0 ± 1.20, and the mean δ^15^N value is 7.3 ± 1.51‰. Dogs from Mas Castellar de Pontós, Sant Esteve d’Olius, and Turó de la Rovira display similar δ^13^C values (ANOVA test: *F* = 1.35; *p* = 0.28). However, concerning δ^15^N values, individuals from Turó de la Rovira exhibit higher values (mean δ^15^N values of 8.47 ± 1.27‰) than those from the other two Iron Age sites, although not statistically significant (ANOVA test: *F* = 4.46; *p* = 0.07). Comparisons can be made between the Iron Age dog remains and those from different periods at Can Roqueta, including the E-MBA, LBA, LBA/EIA, EIA, as well as from the Roman necropolis of Vila de Madrid. The Kruskal-Wallis test reveals substantial differences in both δ^13^C (Kruskal-Wallis test: *H* = 22.11; *p* = 0.001) and δ^15^N values (Kruskal-Wallis test: *H* = 19.56; *p* = 0.003) between the dogs from different levels of Can Roqueta dated between LBA and EIA the ones from Mas Castellar de Pontós, Sant Esteve d’Olius, and Turó de la Rovira (Tables 1h-i). Albizuri and colleagues (2021) hypothesise an intake of C_3_ and C_4_ cultivated plants in the diet of dogs from Can Roqueta, subject to human intervention. Consequently, it appears unlikely that the dogs from the Iberian Iron Age settlements studied here received a significant intake of vegetables. Regarding the mean of δ^15^N values, dogs from Can Roqueta and those from Vila de Madrid appear to have higher values compared to dogs from Mas Castellar de Pontós and Sant Esteve d’Olius and thus more similar to the diet of individuals from Turó de la Rovira.

For *Cervus elaphus* (*n* = 7, all from Sant Esteve d’Olius), the mean δ^13^C value is −21.07 ± 0.52‰, and the mean δ^15^N value is 3.38 ± 0.73‰. The values recorded in the deer from Sant Esteve d’Olius can be compared with those from the Roman site of Carrer Ample 1. The δ^15^N values do not display significant differences between the two sites, reflecting a purely herbivorous diet (ANOVA test: *F* = 0.28; *p* = 0.61). Regarding δ^13^C values, the consumption of C_3_ plants is observed in both cases, albeit with significant differences due to the diverse environment around the two sites (ANOVA test: *F* = 18.26; *p* = 0.001; Rissech et al. 2016).

For *Meles meles* (*n* = 1, from Sant Esteve d’Olius), the δ^13^C value is −18.64‰, and the δ^15^N value is 7.14‰. These values are in line with the ones exhibit by the individual from the EIA of Can Roqueta (δ^13^C value is −18.8‰ and δ^15^N value is 7.6‰; Albizuri et al. 2021).

For the birds (*n* = 15, of which 12 are *Gallus gallus*), the mean δ^13^C value is −19.8 ± 0.92, and the mean δ^15^N value is 8.0 ± 1.13‰. The ANOVA test reveals a significant difference in δ^13^C values between the chickens from the Sant Esteve d’Olius and Turó de la Rovira (ANOVA test: *F* = 18.93; *p* = 0.001). Comparison with chickens from Vila de Madrid and Carrer Ample 1 shows a significant difference in δ^13^C values between the two Roman sites and Turó de la Rovira (Table S1l). On the other hand, the chickens from the two Iberian Iron Age sites display no significant difference in δ^15^N values (ANOVA test: *F* = 2.05; *p* = 0.18), which appear compatible with a diet rich in both plant and animal proteins. This dietary pattern is also observed in chickens from the Roman sites of Vila de Madrid and Carrer Ample 1 (ANOVA test: *F* = 1.08; *p* = 0.37).

Finally, for *Oryctolagus cuniculus* (*n* = 14), the mean δ^13^C value is −21.5 ± 0.83‰, and the mean δ^15^N value is 5.3 ± 2.44‰. Both δ^13^C (ANOVA test: *F* = 0.22; *p* = 0.80) and δ^15^N values (ANOVA test: *F* = 2.31; *p* = 0.15) of rabbits from Mas Castellar de Pontós, Sant Esteve d’Olius, and Turó de la Rovira do not exhibit statistically significant differences. However, some variability in the δ^15^N values is observed among the individuals from Sant Esteve d’Olius and Turó de la Rovira (mean values of 5.9 ± 2.97‰ and 7.0 ± 2.84‰, respectively). The δ^13^C (ANOVA test: *F* = 0.23; *p* = 0.88) and δ^15^N values (ANOVA test: *F* = 1.94; *p* = 0.18) of the two individuals from the Roman site of Vila de Madrid are similar to those of the Iberian rabbits (Salazar-García et al. 2022).

### Sequential δ^13^C and δ^18^O results

**Fig. 3:**
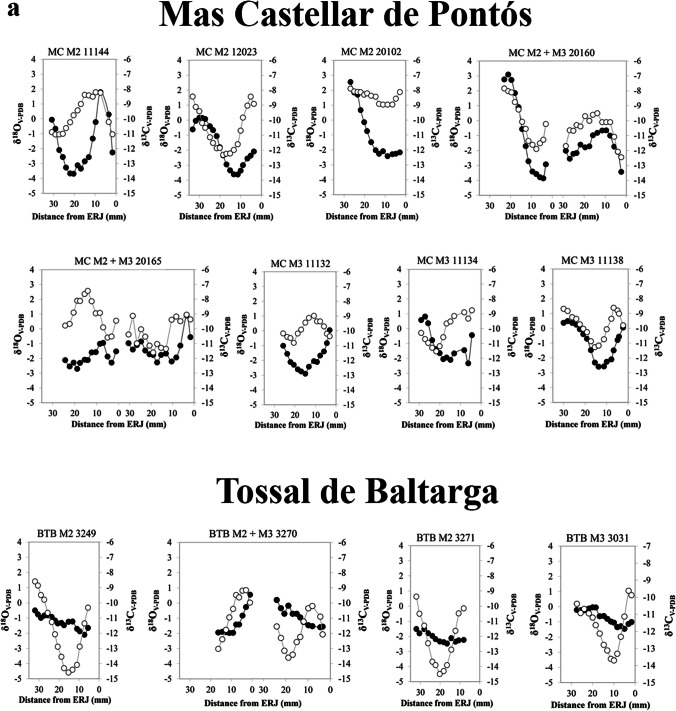

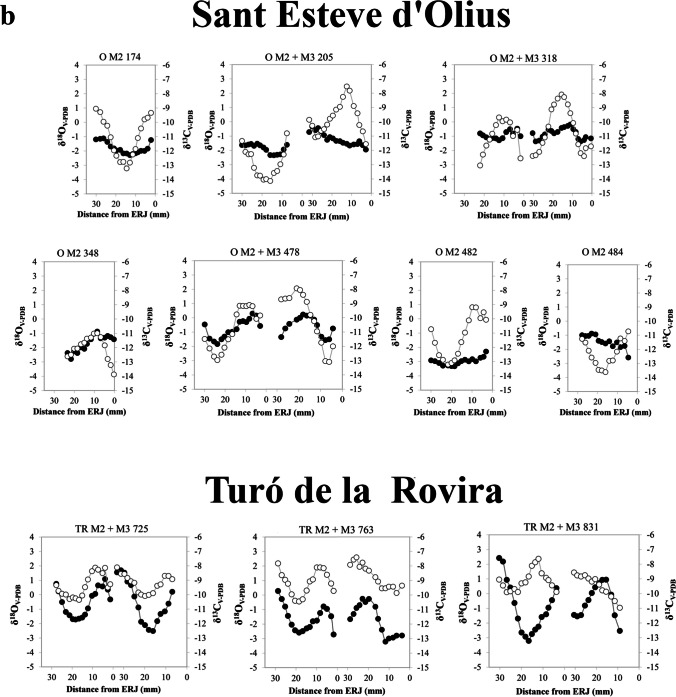
δ^18^O_V−PDB_ (white dots) and δ^13^C_V−PDB_ (black dots) sequences measured on the dental enamel of the second (M_2_) and third (M_3_) lower molars of selected individuals. The M_2_ and M_3_ sequences from the same individuals are presented together. Figure 3a: Mas Castellar de Pontós and Tossal de Baltarga; Fig. 3b: Sant Esteve d’Olius and Turó de la Rovira

Results of the intra-tooth sequences of δ^13^C and δ^18^O values are summarised in Table 4 and fully presented in Fig. 3 and Table S2 The δ^13^C values from all samples range from − 6.9‰ to −13.9‰, with a mean of −11.3 ± 1.14‰. The mean δ^13^C value within each specimen varies from − 10.5‰ to −13.0‰, maximum values range from − 6.9‰ to −12.3‰, and minimum values range from − 11.3‰ to −13.9‰. The δ^18^O values from all samples range from 2.6‰ to −4.6‰, with a mean of 0.4 ± 1.6‰. The mean δ^18^O value within each specimen varies from 1.6‰ to −2.9‰, maximum values range from 2.6‰ to −1‰, and minimum values range from 1.0‰ to −4.6‰.

The sheep from Mas Castellar de Pontós display a sinusoidal pattern and clear maximum and minimum events in both the δ^18^O and δ^13^C sequences, reflecting the seasonal cycle. The second molar of individual MC 20102 is the only exception, with a narrow amplitude of the oxygen sequence. On the other hand, the carbon sequence appears consistent with the others. The δ^18^O sequences from Tossal de Baltarga exhibit high intra-tooth variation along the crown, with a sinusoidal pattern and clear maximum and minimum events. Due to wear, individual BTB 3270 presents an incomplete oxygen sequence, resulting in an incomplete seasonal cycle. In contrast, the δ^13^C sequences of all individuals show low intra-tooth variation. All sheep from Sant Esteve d’Olius display oxygen sequences with great variation along the dental crowns, characterised by a sinusoidal pattern. On the other hand, the carbon sequences display a narrower variation amplitude. At Turó de la Rovira, the sheep δ^18^O and δ^13^C sequences exhibit intra-tooth variations and follow a sinusoidal pattern, with clear maximum and minimum peaks. Due to the young age and consequent incomplete mineralisation of the crown, it is not possible to identify maximum and minimum events in the third molar of individual TR 831. The δ^18^O and δ^13^C sequences included in this study have been previously interpreted in two publications investigating the reproduction and mobility strategies adopted by Iberian Iron Age communities (Messana et al. 2023a, b).

**Table 4:** Summarised oxygen (δ^18^O_V−PDB‰_) and carbon (δ^13^C_V−PDB‰_) values measured on bioapatite samples from the lower second (M_2_) and third (M_3_) sheep molars: mean, range, maximum (max), and minimum (min) isotopic values

Teeth	Sample	*n*	δ^18^O_V−PDB‰_				δ^13^C_V−PDB‰_			
			Mean	Range	Max.	Min.	Mean	Range	Max.	Min.
M_2_	MC 11144	16	0.2	2.8	1.8	−1.0	−11.8	−5.5	−8.2	−13.7
	MC 12023	19	−0.6	3.9	1.6	−2.3	−11.8	−3.8	−9.8	−13.6
	MC 20102	14	1.6	1.1	2.1	1.0	−10.8	−5.0	−7.5	−12.4
	MC 20160	13	0.0	4.1	2.2	−1.9	−10.9	−7.0	−6.9	−13.9
	MC 20165	14	1.0	3.2	2.6	−0.6	−11.9	−1.8	−11.0	−12.7
M_3_	MC 11132	14	0.0	2.3	1.0	−1.2	−11.9	−2.1	−10.8	−12.9
	MC 11134	13	−0.1	2.8	1.2	−1.5	−11.1	−3.2	−9.2	−12.3
	MC 11138	17	0.2	2.7	1.4	−1.3	−10.9	−3.1	−9.5	−12.6
	MC 20160	15	−0.6	2.9	0.5	−2.4	−11.7	−2.8	−10.6	−13.4
	MC 20165	15	−0.3	2.6	0.9	−1.6	−11.3	−3.2	−9.1	−12.3
M2	BTB 3249	16	−1.8	6.0	1.4	−4.6	−11.2	−1.6	−10.5	−12.1
	BTB 3270	10	−0.6	3.9	0.9	−3.0	−11.3	−2.5	−9.4	−12.0
	BTB 3271	13	−2.2	5.1	0.6	−4.5	−12.1	−1.0	−11.5	−12.5
M3	BTB 3031	16	−1.3	4.6	1.1	−3.5	−11.2	−1.3	−10.6	−11.8
	BTB 3270	12	−2.0	7.0	3.4	−3.6	−10.9	−1.8	−9.8	−11.6
M2	O 174	18	−1.1	4.2	1.0	−3.2	−11.8	−1.2	−11.1	−12.3
	O 205	15	−2.9	3.3	−0.8	−4.1	−11.9	−0.8	−11.5	−12.3
	O 318	13	−1.0	3.4	0.3	−3.0	−10.9	−0.8	−10.5	−11.3
	O 348	15	−2.0	2.9	−1.0	−3.9	−11.7	−1.9	−10.9	−12.8
	O 478	16	−0.8	3.9	0.9	−3.0	−10.7	−2.1	−9.7	−11.8
	O 482	16	−1.3	4.0	0.8	−3.2	−13.0	−1.0	−12.3	−13.3
	O 484	14	−2.3	2.9	−0.7	−3.6	−11.5	−1.7	−10.9	−12.6
M3	O 205	19	0.2	4.0	2.5	−1.6	−11.2	−1.6	−10.3	−12.0
	O 318	20	−0.6	4.3	1.9	−2.4	−10.8	−1.2	−10.2	−11.4
	O 478	14	0.0	5.2	2.1	−3.1	−10.6	−1.8	−9.8	−11.6
M2	TR 725	18	0.6	2.2	1.9	−0.4	−10.5	−2.8	−8.9	−11.7
	TR 763	17	0.9	2.6	2.2	−0.4	−11.6	−3.0	−9.7	−12.7
	TR 831	17	0.9	2.3	2.4	0.1	−11.0	−5.6	−7.6	−13.2
M3	TR 725	16	0.8	2.0	1.9	−0.1	−10.5	−4.2	−8.3	−12.5
	TR 763	15	1.3	2.5	2.6	0.1	−11.7	−3.0	−10.3	−13.2
	TR 831	13	0.6	2.4	1.4	−1.0	−10.5	−3.5	−9.1	−12.5

Although the majority of the individuals analysed display δ^13^C values indicative of a diet primarily composed of C_3_ plants, some sheep (MC M2 11144, MC M2 20102, MC M2 20160, and TR M2 831) exhibit evidence of a seasonal contribution of C_4_ plants to their diet. These specimens display δ^13^C values up to −7.7‰ when the oscillating curves of isotopic values reach their maximum peaks along the tooth enamel crown. This suggests a contribution of C_4_ plant not throughout the whole year but during specific and brief periods of life. As previously reported (see Sect. 1.2), the threshold above which δ^13^C values indicate the intake of C_4_ plants is −23‰. To account for the fossil fuel effect, a correction of + 1.5‰ is necessary, resulting in a value of −21.5‰ for pre-industrial plants and − 7.4‰ in the enamel of large ruminant mammals feeding on them.

## Discussion

### Livestock feeding habits

#### *Bone collagen*

The feeding habits of the sheep at the four studied sites appear to be generally homogeneous. The mean and range values of both δ^13^C and δ^15^N show no significant differences. This indicates that this species’ requirements and maintenance must have been similar across the four settlements. Overall, when comparing the results obtained from sheep with those of the other species present in the four Iberian settlements, it is observed that the δ^13^C and δ^15^N values exhibited by sheep fall within an intermediate position relative to those of the other taxa at all sites (Fig. 2). To better understand the feeding habits of the sheep, these are compared below with those of the herbivorous and omnivorous species analysed as a local baseline.

**Fig. 4:**
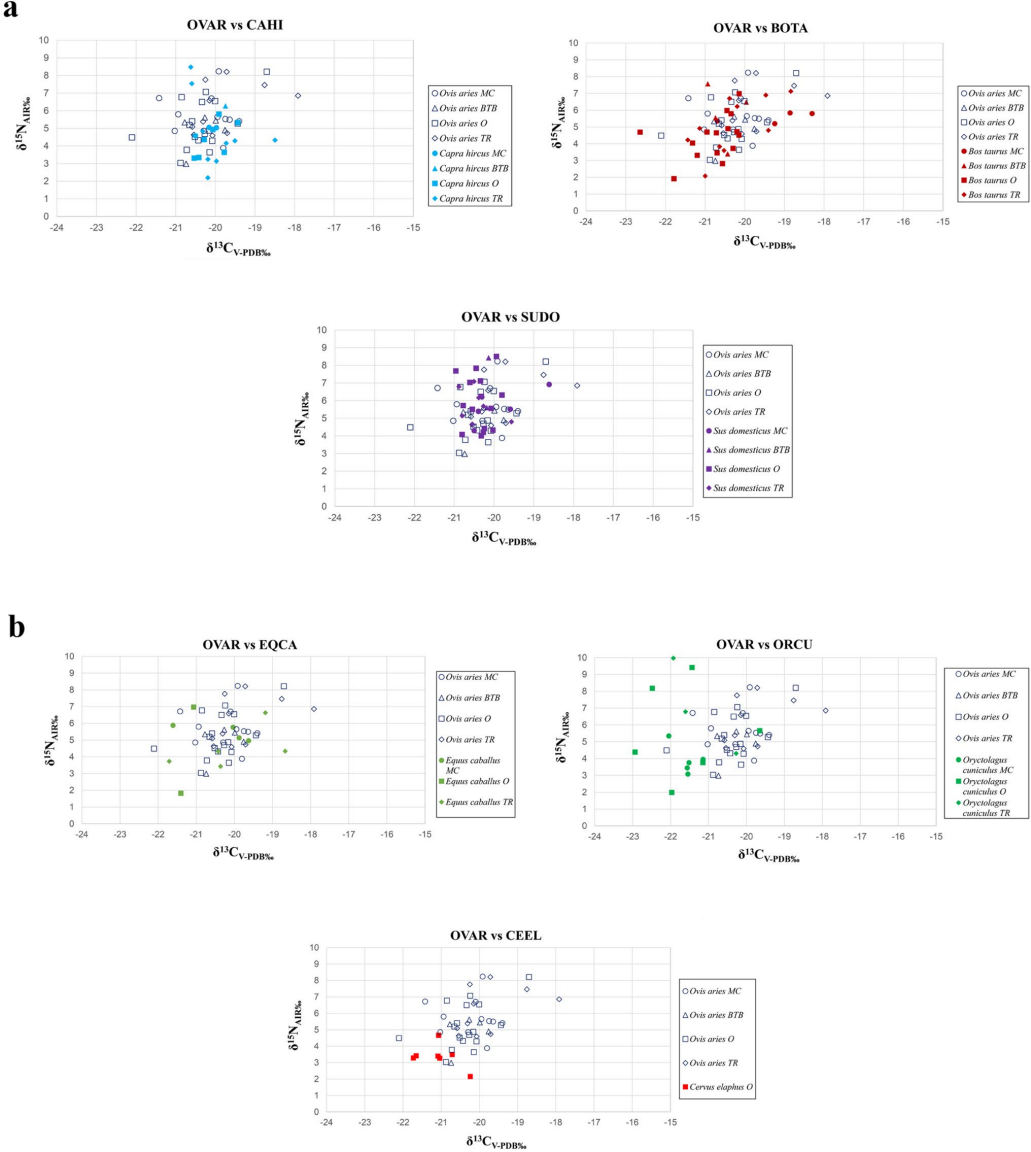
Carbon (δ^13^C_V−PDB‰_) and nitrogen (δ^15^N_AIR‰_) values of bone collagen samples from the four Iberian Iron Age sites. Comparison between sheep and goats, sheep and cattle, and sheep and pigs (Fig. 4a); comparison between sheep and horses, sheep and rabbit, and sheep and deer (Fig. 4b)

Compared to goats, sheep generally exhibit a more varied diet, with higher δ^13^C and δ^15^N values (Fig. 4a). The only exception is the data from Tossal de Baltarga. Unfortunately, only one goat from this site was analysed, which displays higher δ^13^C and δ^15^N values compared to sheep (Fig. 2). While both species consume C_3_ plants, sheep tend to be grazers whereas goats are likely browsers and consume higher vegetation (Papachristou 1997; Bartolomé et al. 1998; Sanon et al. 2007; Dwyer 2009; Jiménez-Manchón et al. 2023). The difference in carbon values between the two species could be attributed to their distinct feeding behaviours.

Sheep and cattle both display similar variability in their δ^13^C and δ^15^N values, which is consistent with the grazing nature of both species (Fig. 4a). However, at Mas Castellar de Pontós the cattle show a different dietary pattern compared to sheep, with higher δ^13^C values suggesting the incorporation of C_4_ plants in their diet (Fig. 2). At Tossal de Baltarga, the sheep diet displays higher δ^13^C values and lower δ^15^N values compared to the cattle. In Sant Esteve d’Olius and Turó de la Rovira, on the other hand, the sheep diet exhibits on average higher δ^13^C and δ^15^N values.

Comparing the results of collagen analyses between sheep and pigs from the four settlements, no significant differences are observed in δ^13^C and δ^15^N values and their variability. However, pigs tend to exhibit less variability in δ^13^C values than sheep (Fig. 4a). At Mas Castellar de Pontós, sheep and pigs display a similar diet, although sheep exhibit slightly lower δ^13^C values on average (Fig. 2). A different picture emerges at Tossal de Baltarga. Here one of the two pigs, an adult, presents considerably higher δ^15^N values (Fig. 2). However, drawing general conclusions on feeding patterns based on data from only two individuals is risky. At Sant Esteve d’Olius, both species show similar δ^13^C values, although sheep display greater variability. Furthermore, pigs exhibit higher δ^15^N values (Fig. 2). Turó de la Rovira sheep present significantly higher δ^13^C and δ^15^N values and a more varied diet compared to pigs (Fig. 2).

Horses from three of the four Iron Age settlements (Mas Castellar de Pontós, Sant Esteve d’Olius, Turó de la Rovira) exhibit a vegetal diet similar to that of sheep, as both are grazing species. However, horses displays lower δ^15^N values compared to sheep (Fig. 4b). It was not possible to analyse the calcined remains of the only horse (BTB EQCA 3224) from Tossal de Baltarga. Sheep from Mas Castellar de Pontós exhibit higher δ^15^N values compared to horses. It is possible that the horses received a supplement of C_4_ cereals in their forage, as indicated by the wide variability and higher δ^13^C values (Fig. 2). The limited number of horses remains analysed at Sant Esteve d’Olius (*n* = 2) prevents us from drawing general conclusions about the feeding management of this species in the Lacetan settlement. Nonetheless, the two individuals show a purely C_3_ plant-based diet, with lower δ^13^C values than the sheep. Overall, sheep exhibit higher δ^15^N values, although the two horses display completely different δ^15^N values relative to one another (Fig. 2). At Turó de la Rovira, sheep show less variability in their vegetal diet and higher δ^15^N values compared to the horses (Fig. 2).

Sheep and rabbits exhibit significantly different vegetal diets. Rabbits from the three Iberian sites (no remains are available for Tossal de Baltarga) have much lower δ^13^C values than sheep, indicating the consumption of plants and resources from dense forest habitats. Although the δ^15^N values are similar for both animals, rabbits show greater variability within sampled sites (Fig. 4b). At Mas Castellar de Pontós, sheep display higher δ^13^C and δ^15^N values (Fig. 2). At Sant Esteve d’Olius and Turó de la Rovira, sheep show higher δ^13^C, whereas rabbits exhibit higher δ^15^N values and greater variability in ^15^N content (Fig. 2).

When comparing the feeding habits of the sheep from the four Iron Age sites with those of the deer from Sant Esteve d’Olius, it is evident that sheep display a more variable diet with higher δ^13^C and δ^15^N values (Fig. 4b). The same trend is observed within the Lacetan settlement, confirming the distinct diets of the two species and probably suggesting differences between wild and domestic grazer herbivores (Fig. 2).

#### *Dental enamel*

Sheep feeding habits, previously analysed through the δ^13^C values resulting from bulk collagen analysis, are now further interpreted by considering the δ^13^C values obtained from the sequential analysis of dental bioapatite. The sequential analysis confirms that the sheep primarily consumed C_3_ plants. However, it reveals seasonal variability in the carbon values of the four settlements, in contrast to the general homogeneity observed in the sheep’s diet through collagen analysis (Fig. 3). Indeed, along the dental crown, the δ^13^C values indicating consumption of C_4_ plants by individuals coincide with the duration of the maximum δ^18^O values recorded during the warm season. As a result, based on the variability and amplitude of the carbon sequences, the four sites can be differentiated into two groups, with individuals from Mas Castellar de Pontós and Turó de la Rovira exhibiting wider ranges compared to individuals from Tossal de Baltarga and Sant Esteve d’Olius. The carbon sequences of the first group (MC and TR) are generally wide and closely mirror the fluctuations of the oxygen sequences, which are indicative of seasonal changes. These variations result from a feeding regime influenced by seasonal climatic variability and the resulting availability of plant resources throughout the year. In this context, plants experience water stress during warmer periods, leading to higher δ^13^C values, which coincide with higher δ^18^O values. In the second group (BTB and O), the δ^13^C sequences generally display a very narrow amplitude, while the oxygen sequences exhibit seasonal variations. This suggests that feeding in these settlements was not strongly subject to seasonally-based plant consumption. Therefore, it is plausible that the sheep’s diet was supplemented with additional fodder and water during periods when pasture resources were limited.

At Mas Castellar de Pontós, the eight individuals analysed generally had a diet primarily based on C_3_ plants. However, three individuals - MC 11144, MC 20102, and MC 20160 –show a different pattern. We know that individual MC 11144 was born in spring, whilst individuals MC 20102 and MC 20160 were born in autumn (Messana et al. 2023a). The analysis of the δ^13^C sequence obtained from the M_2_ of individual MC 11144 suggests that C_4_ plants contributed to its diet during its second summer alive (Fig. 3). However, since there is no information available about the individual’s first summer alive due to the crown wear, it is unclear whether this supplement of C_4_ plants was a systematic and repetitive seasonal practice or a punctual or sporadic event. In both individuals MC 20102 and MC 20160, there is evidence of a contribution of C_4_ plants in their diet during their first summer (Fig. 3). For individual MC 20160, it was also possible to analyse its M_3_ and the δ^13^C sequence displays a reduced amplitude and values consistent with a C_3_ plant-based diet. Therefore, our data show that C_4_ plants were integrated into the diet of individual MC 20160 on a punctual basis rather than seasonally. It appears that at Mas Castellar de Pontós the feeding strategies for sheep included the occasional contribution of C_4_ plants, likely cereals, to the fodder when grazing resources were limited (i.e. summer peak). Nevertheless, the possibility that the C_4_ plants introduced into the sheep’s diet were wild cannot be entirely ruled out. Indeed, wild C_4_ plants, particularly halophytes, are attested in the coastal marshes area of the Empordà plain, especially in the Gulf of Roses, and may have represented a feed resource during the dry season (Houérou 1994; Gestí 2006; Casals 2007; Ejarque et al. 2016). However, wild halophytes have a high salt content and present various anti-nutritional factors, which limit their consumption by livestock (Houérou 1994; El Shaer and Attia-Ismail 2002; Stringi et al. 2009; Abd El-Hack et al. 2018; Hasnain et al. 2023). Moreover, they are more digestible and nutritious during wet seasons than during dry seasons (El Shaer and Attia-Ismail 2002; El Shaer 2006). Tracing this dietary supplement is more challenging when considering the values obtained from bulk collagen analyses carried out on bone remains. For instance, the δ^13^C value obtained from the mandible of individual 20160 is −19.91‰, which provided an attenuated signal of the C_4_ plants’ contribution to the diet in its later years (Table 1). The same individual also displays particularly high δ^15^N values (8.24‰). The higher δ^13^C and δ^15^N values may suggest the intake of crops cultivated in fertilised fields or the consumption of halophyte plants from coastal salt marshes (Bogaard et al. 2007; Britton et al. 2008; Fraser et al. 2011; Müldner et al. 2014; Szpak 2014; Guiry et al. 2021; Makhad et al. 2022). However, no conclusions can be drawn based on a single individual. Similar δ^15^N values around − 19‰ are observed in the bone collagen of five other individuals, suggesting that they might have consumed C_4_ plants at some point in their lives, but seasonal contribution could be masked in multi-year values.

In the Ceretan settlement of Tossal de Baltarga, the carbon sequences of three of the four sheep individuals analysed - BTB 3031, BTB 3249, and BTB 3271 - exhibit a very narrow amplitude, indicating minimal to no variations in the diet on a seasonal basis. Regarding individual BTB 3270, after its second summer, a simultaneous increase in carbon values and a decrease in δ^18^O values is observed. This results in an inverse relationship between the two sequences. The increase in δ^13^C values corresponds to the consumption of ^13^C-enriched plants in a drier environment, suggesting a change in pastures due to an altitudinal movement to lowlands areas. After the second winter, δ^13^C values decrease as δ^18^O values increase, indicating another change in altitude and feeding habits, and a return to the original location (Messana et al. 2023b). In this case, the increase in δ^13^C values is not due to the contribution of C_4_ plants in the forage. The sequential analyses on the dental bioapatite are consistent with the results obtained from the bulk collagen analyses, confirming a purely C_3_ diet for the sheep.

At Sant Esteve d’Olius, the δ^13^C values from the seven sheep individuals analysed depict a herd primarily fed on C_3_ plants, with no significant seasonal variations in diet. Even individual O 205, which moved from a different location to Olius during its third winter, displays a similar pattern of the δ^13^C sequence, with a limited amplitude in its early years (Messana et al. 2023b). The sequential analysis results are consistent with those obtained from the bulk collagen analysis, except for individual O 484. For the latter, the collagen analysis indicates a contribution of C_4_ plants to its diet, which is not reflected in the sequential analysis. This suggests that the dietary integration in individual O 484 must have occurred after the mineralisation of its second lower molar. Furthermore, the high δ^15^N value in this individual could suggest the consumption of cereals grown in manured fields, as previously hypothesised for individual 20160 from Mas Castellar de Pontós.

Finally, two out of the three sheep individuals from Turó de la Rovira - TR 725 and TR 763 - both born in spring, exhibit wide and seasonally fluctuating δ^13^C sequences. On the other hand, the third individual - TR 831 - born at the beginning of summer, practised short-range seasonal mobility along a low altitudinal gradient (Messana et al. 2023a, b). Indeed, after its first winter, an increase in δ^18^O values coincides with a decrease in δ^13^C values, indicating a movement towards a wetter environment and a corresponding change in diet. After the second summer, another change in the pasture occurs, which is reflected in a decrease in δ^18^O values and a simultaneous increase in δ^13^C values (Messana et al. 2023b). Therefore, the change in feeding is due to a seasonal change in pastures. Specifically, individual TR 831 fed on wetter pastures during its second summer, before returning to Turó de la Rovira and feeding there during its second winter. An additional noteworthy aspect is the recording of high δ^13^C values towards the end of individual TR 831’s first summer, suggesting a potential contribution of C_4_ plants to its diet, although data is not conclusive at all. Previous isotopic studies on reproduction strategies and livestock mobility at Turó de la Rovira suggest that there was a differentiated flock management and, probably, a division of the herd into two distinct groups. The data presented in this study reinforce this idea, further articulating the livestock management strategies adopted in the Laietan settlement. Part of the flock, specifically those born in the spring, would have remained in Turó de la Rovira year-round. On the other hand, the individuals born during summer, starting from their second year of life, would have joined the practice of seasonal altitudinal mobility. Among this latter group, the newborns that were too young to undergo seasonal migration, would have stayed in the settlement and received a supplement of C_4_ plants into their fodder. The latter probably consisted of cereals, although the hypothesis that it comprised wild C_4_ plants from coastal salt marshes cannot be ruled out. Indeed, the presence of halophyte plants, albeit minimal, is attested in the Penedès area and especially in the Llobregat delta (Pla de Ports de Catalunya 2007–2015; Valenzuela-Lamas et al. 2016; Salazar-Mendías and Lendínez 2020). This management, including the migration of only a part of the flock and dietary supplements, might have been influenced by the limited availability of pastures during the dry summer season. However, the limited number of individuals analysed hinders in-depth deductions. The collagen analysis performed on the remains of ten sheep suggests a diet mainly consisting of C_3_ plants. Nevertheless, the δ^13^C value obtained from the mandible of individual TR 831 (−18.76‰) could indicate the integration of C_4_ plants in its diet. Moreover, the slightly higher δ^15^N value (7.46‰) of this individual may support both the hypothesis of the consumption of cereals from manured fields as well as of halophyte plants from coastal salt marshes. Individual 763 displays a higher δ^15^N value (8.21‰), but a lower δ^13^C value (−19.72‰). On the other hand, an even higher δ^13^C value (−17.91‰) is observed in individual TR 819, suggesting the consumption of C_4_ plants during its lifetime. Unfortunately, neither M_2_ nor M_3_ were recovered from this individual, making it impossible to verify if it underwent a similar management as individual TR 831.

### Livestock feeding management

The data obtained from the collagen isotope analysis shed new light on the diversified livestock feeding strategies adopted by Iberian communities during the Middle/Late Iron Age. Particular focus was reserved for sheep and caprines in general, which were the most prevalent species in the four settlements.

The homogeneous feeding pattern observed for sheep in the four studied settlements reveals the particular care given to the management of this species. According to Columella in Book VII of *De re rustica*, the Greek philosopher Celsus affirmed that sheep are a very delicate species, confirming the special attention reserved for them by shepherds (VII.II.2). In addition, the Roman author advises feeding them in abundant grassy pastures, while Varro recommends year-round outdoor grazing for the flock (Colum. VII.III.9; Varro II.II.7). Both authors agree on the importance of keeping sheep away from shrubs and bushes to prevent damage to their fleece. The results of the sequential analysis of second and third molars from sheep paint an even more intricate picture of their seasonal feeding management. Although limited to approximately the first two years of an individual’s life, this analysis offers a highly accurate yearly and seasonal resolution. The north-eastern Iberian Peninsula is characterised by the Mediterranean climate, known for its high variability and marked seasonal fluctuations in pasture availability and rainfall (Davies 1941; Ruiz and Ruiz 1986; Oteros-Rozas et al. 2013). Therefore, it becomes crucial to detect, at a seasonal scale, the livestock feeding strategies adopted by Iron Age Iberian shepherds to adapt to the Mediterranean basin’s fluctuating climatic conditions. Additionally, the integration of crop residues into the livestock diet likely adhered to a seasonal pattern tied to their availability. The possible use of these animals for field clearance between sowing seasons cannot be excluded. Finally, seasonal variations in feeding habits could be related to forage supplementation for pregnant females or males preparing for mating. In *De re rustica*, Book II, Varro states that rams should be fed more fodder and barley two months before mating to strengthen them (II.II.13). Therefore, it cannot be ruled out that cereal integration in an individual’s diet might also depend on these factors. However, as previously mentioned, the hypothesis that sheep from Mas Castellar de Pontós and Turó de la Rovira may have consumed wild C_4_ plants from coastal salt marshes should not be dismissed. With the currently available data, it seems that the feeding habits of sheep appear to be similar not only among the Iberian communities but also in later periods, as suggested by the analysis carried out in the Roman necropolis of Vila de Madrid (Salazar-García et al. 2022).

It is plausible that sheep and goats shared pastures within the same flock. However, the two species differ in their dietary preferences. Goats primarily consume C_3_ plants in all four settlements and display a less diversified diet, with lower δ^13^C and δ^15^N values compared to sheep. Columella and Varro point out that goats are more inclined to pasture in rugged areas and wooded glades, consuming shrubs and brambles (Colum. VII.VI.1; Varro II.III.6–7). In addition, Varro highlights the behavioural differences between the two caprine species, with sheep exhibiting a docile and gregarious nature, whereas goats are inclined to disperse and move around in impervious environments (Varro II.II.3). Therefore, it is conceivable to imagine a mixed herd in which both species, although pasturing in proximity, have access to distinct plant resources. Finally, the exploitation of different products from both species should also be considered. While sheep were primarily exploited for wool, goats were mainly exploited for milk and its derived products such as cheese. The different feeding strategies employed by herders may correspond to diverse livestock practices and exploitation. The generally lower δ^15^N values observed in goats compared to sheep in all four sampled sites could suggest a legume supplement in their diet. Indeed, legumes provide excellent hay and their nutrient and plant protein content can have positive effects on milk production and composition (Morand-Fehr and Sauvant 1980; Harris et al. 1996; Ahmed and Nour 1997; Bonanno et al. 2013).

The results obtained from the analysis of cattle remains reveal a general similarity in diet with sheep. However, when considering the data obtained from each site, the diet of the two species differs. Furthermore, the cattle diet varies between the different settlements. Therefore, Iron Age Iberian herders employed diverse feeding strategies for cattle between settlements. Moreover, within the same settlement, they fed caprines and cattle with different types of fresh vegetation and/or fodder. Indeed, the dietary requirements of each animal may differ according to species, age, sex, and intended use. Therefore, the differences observed in the diets of caprines and cattle within the same settlement, as well as among cattle from different settlements, are not unexpected. However, this livestock management approach implies the employment of specialised and tailored feeding strategies for each species reared in the settlement, along with the availability of diverse pastures and forage resources to suit the specific needs of each animal. The introduction of C_4_ plants in the cattle diet at Mas Castellar de Pontós suggests a supplement of cultivated cereals in their forage. The integration of C_4_ plants into the livestock diet in the rural settlement is well attested by the results obtained through sequential isotope analysis on sheep teeth. Moreover, the presence of both winter and spring crops within the settlement would have guaranteed the availability of crop residues for a significant portion of the year, particularly during seasonal periods when grazing resources were limited (Canal 2000, 2002). Columella mentions the use of cereal chaff for cattle feed, emphasising millet as the most prevalent option, followed by barley and wheat (VI.III.3). The extensive presence of millet, a C_4_ cereal, within the rural establishment, supports the plausibility of its utilisation for feeding the cattle of Mas Castellar de Pontós (Canal 2000, 2002). However, Columella and Varro recommend grazing cattle in grassy, well-watered pastures (Colum. VI.III.2; Varro II.V.14). Moreover, in drier areas, Columella suggests feeding them with legumes and hay meadows within the stables (VI.III.3). Nevertheless, as in the case of sheep, the hypothesis that the integration of C_4_ plants into the cattle’s diet resulted from the consumption of halophyte plants from coastal salt marshes should not be rejected. At Tossal de Baltarga, both cattle and sheep were primarily fed on C_3_ plants. However, the cattle’s vegetal diet appears more homogeneous compared to those of sheep. This may depend on the frequentation of different pastures for the two animals: cattle grazed in open pastures, whereas sheep pastured in the wooded areas surrounding the settlement, consuming a more heterogeneous variety of plants. The cattle from Sant Esteve d’Olius had a diet very similar to that of sheep. However, some individuals exhibit values suggestive of having consumed plants from closed-canopy forests. According to Varro in Book II of *De re rustica*, cattle graze in wooded areas and consume undergrowth and foliage (II.V.11). Therefore, a non-generalised contribution of these plants to their diet within the Lacetan settlement cannot be ruled out. At Turó de la Rovira, the cattle feeding habits were similar to those of sheep. Nevertheless, their diet manifested a higher degree of variability. Some individuals consumed small add-ons of plants from wooded areas, while others included C_4_ plants in their diet. It is feasible that the cattle were allowed to graze unrestricted around the Laietan settlement, feeding on both cultivated and uncultivated terrains. With all these data, we can propose that the feeding strategies adopted by Iron Age Iberian herders for cattle varied from one settlement to another according to the requirements of each community. In contrast, the few collagen results available for the Bronze Age and Early Iron Age settlements in the north-east of the Iberian Peninsula revealed a more homogeneous scenario, with the integration of C_4_ plants into the cattle’s diet (Grandal-d’Anglade et al. 2019; Albizuri et al. 2021). On the other hand, the cattle feeding habits during the Roman period indicate a diet primarily based on C_3_ plants (Rissech et al. 2016; Salazar-García et al. 2022). The data obtained from the four Iron Age sites are thus in an intermediate position between the earlier and later periods, demonstrating the great adaptability and variability of Iberian communities in livestock management.

In the four Iron Age settlements, the pigs’ alimentation primarily consisted of C_3_ plants, which aligns with the dietary patterns of sheep. Additionally, their not generally high δ^15^N values suggest a mildly omnivorous diet. Slightly higher δ^15^N values are observed in pigs from Sant Esteve d’Olius. However, the δ^15^N values suggest that the diet was still predominantly herbivorous, probably with minimal contribution from culinary waste. It is possible that pigs were allowed to graze freely or semi-freely in the outdoors, rather than reared in enclosures with a controlled diet. Both Columella and Varro mention the use of legumes as pig fodder (Colum. VII.IX.9; Varro II.IV.6). Their inclusion in suids’ diet could have contributed to the generally low δ^15^N values observed, as possible signs of omnivorous behaviour might have been attenuated by legume consumption. A previous study by Lösch and colleagues (2006) provided support for this hypothesis suggesting the consumption of legumes by sheep, goats, and pigs from the Pre-Pottery Neolithic B site of Nevali Çori to explain the lower δ^15^N values recorded in these taxa. On the other hand, the available data on Bronze and Early Iron Age Iberian pigs indicates a feeding strategy rich in ^15^N (Grandal-d’Anglade et al. 2019; Albizuri et al. 2021). In contrast, the few data available from the Roman Period suggest that pig dietary habits experienced a significant shift, resulting in generally lower but more varied δ^15^N values (Rissech et al. 2016; Salazar-García et al. 2022). Therefore, as in the case of cattle, the data from Iron Age Iberian settlements related to pigs’ feeding strategies falls into an intermediate position between earlier and later periods.

This study documents the seasonal consumption of C_4_ plants by sheep at Mas Castellar de Pontós. This feeding habit is also observed in cattle and probably horses, but it is not possible to determine whether the consumption was continuous or sporadic for these two species. The results of both bone collagen and sequential dental analyses suggest a supplement of C_4_ plants also in the diet of cattle from Turó de la Rovira. Therefore, it is evident that Iron Age Iberian shepherds supplemented livestock’s diet with C_4_ plants, whether cultivated cereals or wild plants, according to their necessity.

The integration of C_4_ plants into livestock diet was not common during prehistoric and protohistoric times in the north-east or the entire Iberian Peninsula (Navarrete et al. 2019, 2023, 2024; Tejedor-Rodríguez et al. 2021; Martín et al. 2021; Sierra et al. 2021). Nevertheless, it is important to consider the geographic position of the Iberian Peninsula, which is located in the southernmost area of Europe. This may have provided opportunities for C_4_ plants to thrive in drier ecological niches. Regarding protohistoric times in the north-eastern Iberian Peninsula, Albizuri and colleagues (2021) suggest that dogs from the Can Roqueta site, dated between the Late Bronze Age and the Early Iron Age (1300 and 550 cal. BC), consumed C_4_ cereals (millet), following the human diet. Furthermore, the data obtained from foxes also indicate consumption of C_4_ cereals, suggesting a commensal relationship on the part of foxes during the Bronze Age. Finally, cattle also display values compatible with the integration of C_4_ plants into their diet. Therefore, it cannot be ruled out that herbivorous species occasionally or seasonally consumed these plants. Further research could expand our understanding of the consumption of C_4_ plants by sheep and other livestock, providing a more comprehensive view.

## Conclusion

The data from this study reveal for the first time the livestock feeding strategies adopted by Middle/Late Iron Age communities in the north-eastern Iberian Peninsula. The bulk collagen analysis on the bone remains of the main taxa from four Catalan sites and the sequential analysis in the second and third lower molars of sheep have unveiled the complex, diversified, and adaptive nature of the husbandry practices employed by Iron Age Iberian populations. Sheep, the predominant species during the Iron Age, were provided with specific care and a homogeneous diet, mainly consisting of C_3_ plants at all four sites. However, their feeding habits were subject to seasonal changes depending on pasture availability and settlement requirements. As a result, different patterns of sheep diet management were observed in the four settlements studied. At Mas Castellar de Pontós and Turó de la Rovira, the seasonal climatic variability and the rarefaction of pastures during the summer influenced the feeding habits of the herds. Additionally, during the summer months, the sheep’s diet at both settlements was supplemented with C_4_ plants. Herd feeding strategies at Tossal de Baltarga and Sant Esteve d’Olius appeared less reliant on seasonal variations in pasture availability. There probably was human intervention in sheep feeding, with the contribution of C_3_ fodder and water when pasture resources were limited, especially during winter.

The data obtained from the other main domestic species in the four settlements (goats, cattle, and pigs) have expanded and enhanced the knowledge of livestock husbandry practices employed by the Iron Age Iberian communities. Furthermore, the integration of results from bulk collagen and sequential analyses performed on sheep remains allowed a more comprehensive understanding of the carbon isotopic composition of collagen from other domestic taxa. The results reveal a complex husbandry system in which, within the same settlement, each species was subject to individualised feeding strategies. We suggest that these varied not only depending on the needs of each animal but also in response to the availability of pasture and fodder, as well as the demands for secondary products specific to each community.

Therefore, this degree of labour organisation suggests that animal husbandry played a more crucial role in the economy of the Iron Age Iberian communities than it has been given so far. Hence, it is plausible that there was a division and specialisation of activities within the settlements, with agriculture on one side and animal husbandry on the other. Nevertheless, these two systems were interlinked and perfectly integrated into their respective schedules.

The results obtained on livestock feeding strategies are consistent with those related to reproduction strategies and mobility of sheep presented in Messana et al. (2023a, b). Indeed, the different husbandry practices employed by the Iberian communities are also reflected in the distinct patterns of both sheep reproduction and mobility in the four settlements.

On a methodological level, this study provides a more comprehensive insight into the feeding strategies of sheep through the combination of isotopic analyses on the organic fraction of bone remains (bulk collagen) and the mineral fraction of dental remains (sequential analysis on enamel bioapatite). The sequential analysis of δ^18^O and δ^13^C allowed the reconstruction of sheep’s dietary habits during approximately their first two years of life, with a seasonal resolution. This level of detail is inevitably forfeited through δ^13^C and δ^15^N analysis on bulk collagen. Nevertheless, this analysis revealed the protein sources, whether of plant or animal origin, consumed by the main species, sheep including, in the four Iron Age Iberian settlements during approximately their final decade of life. Therefore, both analyses proved to be complementary and necessary for a complete understanding of the livestock feeding strategies adopted by Iberian shepherds and the reasons guiding their adoption.

This research highlights the adaptive nature of the Iron Age Iberian populations and their ability to manage their livestock in a way that maximises their resources while meeting each animal’s specific needs. Furthermore, the carbon and nitrogen values from cattle and pigs respectively occupy an intermediate position between those available in the literature for the Bronze Age to Early Iron Age and the Roman period. Thus, based on the currently available data, there does not appear to have been an abrupt change in the feeding strategies of these two species between the Iron Age and the Roman period. Finally, the results presented in this study constitute the first data on livestock feeding strategies for the Middle/Late Iron Age in the north-eastern Iberian Peninsula. The extensive number of remains and the variety of species analysed provide the first baseline for interpreting δ^13^C and δ^15^N results for this chronology and area. The results obtained demonstrate that it is imperative to conduct further research on additional Iron Age Catalan settlements, as well as Roman sites, to investigate the evolution of livestock feeding strategies during the transition between these two periods. Furthermore, the crucial relevance and considerable potential of isotopic analyses for a broader understanding of the economic and social dynamics within protohistoric societies is evident.

## Supplementary Information

Below is the link to the electronic supplementary material.

ESM1 (XLSX 33.2 KB)

ESM2 (XLSX 25.6 KB)

## Data Availability

No datasets were generated or analysed during the current study.
